# *In situ* insights into antibody-mediated neutralization of a pre-fusion Junin virus glycoprotein complex

**DOI:** 10.1016/j.celrep.2025.115971

**Published:** 2025-07-08

**Authors:** Lily J. Taylor, Michael R. Sawaya, Jonna B. Westover, Chenyi Wang, Frederick Jimenez, Aldo J. Muñoz, Julian Whitelegge, Brian B. Gowen, Gustavo F. Helguera, Roger Castells-Graells, Jose A. Rodriguez

**Affiliations:** 1 Department of Chemistry and Biochemistry, UCLA-DOE Institute for Genomics and Proteomics, STROBE, NSF Science and Technology Center, University of California, California, Los Angeles (UCLA), Los Angeles, CA 90095, USA; 2 Department of Animal, Dairy and Veterinary Sciences, Utah State University, Logan, UT 84322, USA; 3 The Pasarow Mass Spectrometry Laboratory, The Jane and Terry Semel Institute for Neuroscience and Human Behavior, David Geffen School of Medicine, University of California, California (UCLA), Los Angeles, CA, USA; 4 Laboratory of Pharmaceutical Biotechnology, Instituto de Biología y Medicina Experimental (IBYME-CONICET), Buenos Aires, Argentina; 5 Present address: Structural Biology Programme, Spanish National Cancer Research Centre (CNIO), Madrid, Spain; 6 Lead contact

## Abstract

A transmembrane glycoprotein complex (GPC) decorates the Junin mammarenavirus (JUNV) that causes New World hemorrhagic fevers. We leveraged single-particle cryoelectron microscopy (cryo-EM) to image the full-length JUNV GPC directly on pseudotyped virus (PV) membranes and bound by two JUNV-neutralizing antibodies: Candid#1 vaccine-elicited CR1–28 and J199, a potent therapeutic against Argentine hemorrhagic fever (AHF). The 3.8 Å resolution *in situ* structures of the antibody-neutralized, 3-fold symmetric JUNV GPC reveal its ectodomain architecture, signal peptide-bound transmembrane region, zinc-binding luminal domain, and post-translational modifications. JUNV-GPC sequence variants highlight the functional importance of the signal peptide transmembrane helix register for virus infection and attenuating Candid#1-associated variants. Overlapping CR1–28 and J199 epitopes suggest a common receptor-blocking mechanism for JUNV neutralization, while a J199-induced, symmetric GPC reorientation may further drive its potent inhibition of JUNV lethality in mice, compared to receptor blockade alone. This underscores the utility of *in situ* insights into GPC function and neutralization.

## In brief

Taylor et al. use single-particle cryo-EM to determine the *in situ* structure of a Junin virus glycoprotein complex (GPC). Structures of GPC-bound neutralizing antibodies, CR1–28 and J199, uncover molecular details of their GPC neutralization and their potential stabilization of a pre-fusion GPC conformation.

## Graphical Abstract

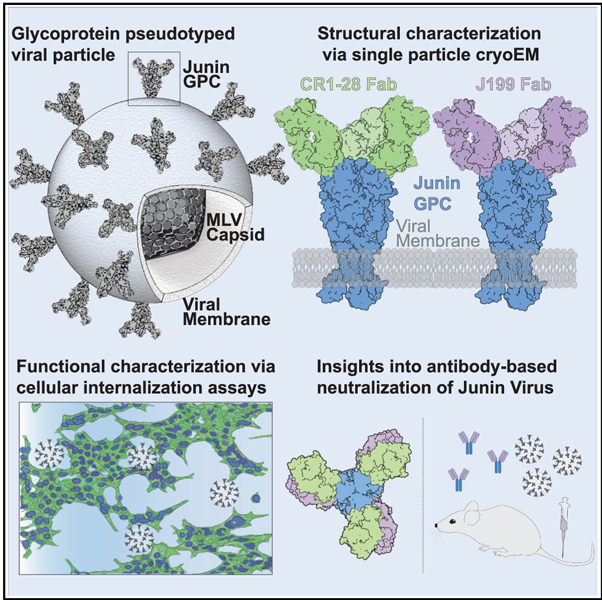

## INTRODUCTION

**Figure 1.**
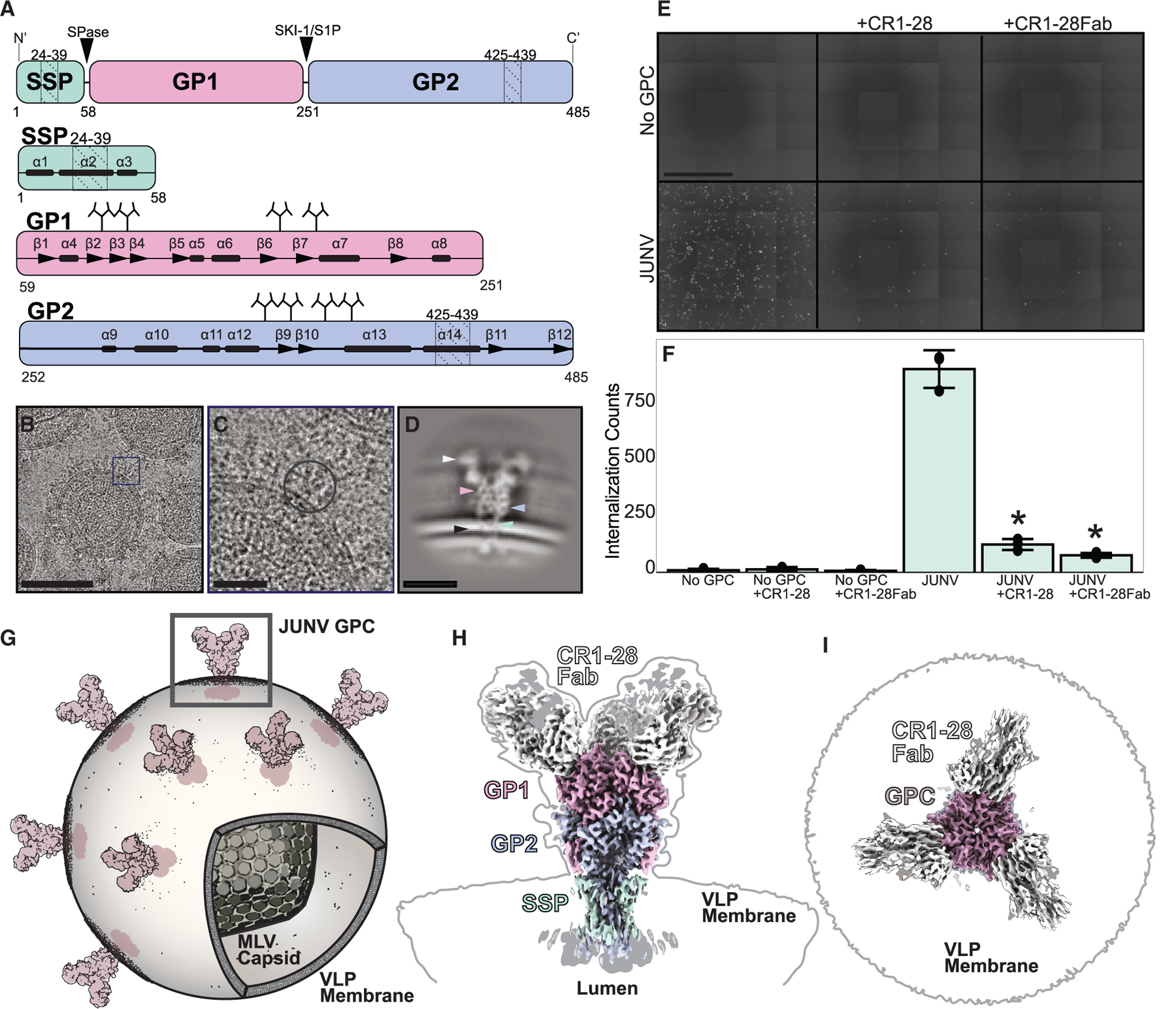
*In situ* structural analyses of an antibody-neutralized Junin GPC (JUNV) on a PV membrane (A) Domain organization of the JUNV GPC, depicting N-linked glycosylation sites (tree diagrams, UniProt: P26313), transmembrane regions (dotted lines), α-helical regions (bars), and β strands (arrows). (B) Cryo-EM micrograph of the CR1–28Fab-bound JUNV GPC. Scale bar, 100 nm. (C) Magnified view of box in (B), with single GPC on the virus membrane selected for processing. Scale bar, 25 nm. (D) Representative 2D class of CR1–28Fab-bound JUNV GPC. Scale bar, 10 nm. Arrows point to structural regions of the CR1–28Fab-bound JUNV GPC: white, CR1–28Fab; pink, GP1; blue, GP2; green, SSP; black, MLV membrane. (E) Images showing GFP fluorescence in HEK293T cells as a readout for internalization of JUNV GPC PVs used for cryo-EM, with CR1–28 and CR1–28Fab present at 100 μg/mL. Scale bar, 1 mm. (F) Quantification of GFP signal in (E). Data are represented as mean total GFP counts for each condition ± standard deviation. *p < 0.01, comparing antibody-inhibited JUNV PV internalization relative to uninhibited JUNV PV internalization determined by Welch’s t test. *n* = 3 technical replicates for each condition. (G) Schematic of Fab-bound JUNV GPC on MLV particle; the box selects a single GPC. (H and I) Side (H) and top (I) views of cryo-EM maps rendering a JUNV GPC at various thresholds: 1 (color), 0.5 (gray), and 0.2 (black outline). See also Figures S1–S7, S15, and S20 and Table S1.

New World mammarenaviruses (NWMs) are enveloped zoonotic agents that can cause human hemorrhagic fevers^1^; they include Machupo (MACV), Guanarito (GTOV), Sabia (SABV), Chapare (CHAV), and Junin (JUNV) viruses. NWMs exist as quasispecies and contain a bisegmented, negative-sense, single-stranded RNA genome whose small (S) segment encodes for a nucleoprotein and a glycoprotein precursor polypeptide.^2^ That precursor is processed by the human peptidases SPase and subtilase SK1/S1P into three polypeptides^3–5^: a stable signal peptide (SSP), a receptor-binding domain (GP1), and a membrane-associated domain (GP2)^6,7^ (Figures 1A and S1). Three copies of each non-covalently associate as part of a mature, 3-fold symmetric homotrimeric membrane integral glycoprotein complex (GPC) that alone decorates the surface of the NWM envelope.^8–10^

The overall domain architecture of the Junin GPC resembles those of other NWMs, including Machupo, Guaranito, and Sabia,^11–13^ and shares similarities with the Old World viruses Lassa and Lujo^14,15^ (Figure S1). Proper assembly, trafficking, and installment of those GPCs on the outer membrane of NWMs are dependent on the cleavage of their SSP from the nascent polypeptide and on its association with the transmembrane region of GP2.^1,6,16,17^ Heterologous expression of the SSP can facilitate proper GPC assembly and installment on recombinant JUNV strains, but its exact interface with GP2 and the *in situ* topology of the GP2-SSP transmembrane complex remain unclear.^17,18^ Cell fusion and pseudotyped virus (PV) assays have identified the direct role of SSP-GP2 interactions in the pathogenicity of NWMs,^18,19^ underscored by the epistatic effects of single site variants across the two domains.^20^ Those interactions govern the structure and dynamics of the GPC in the membrane and are essential for understanding its role in mammarenavirus function.^12,18,21,22^

Structures of isolated Old World mammarenavirus and NWM GPCs and their components have illuminated their receptor engagement and targeting by therapeutics.^9,13–15,21,23^ Crystallographic structures have revealed the Machupo GP1 interface responsible for human transferrin receptor 1 (hTfR1) binding, which is shared by JUNV and other pathogenic NWMs.^9,24^ Structures of GP1-targeting antibodies have revealed distinct modes of neutralization, including those adopted by JUNV- or MACV-specific antibodies and JUNV-MACV cross-reactive antibodies, indicating that broader neutralization may be possible by targeting epitopes on GP1 within its receptor-binding domain.^25,26^ However, despite these efforts, pathogenic NWMs lack FDA-approved clinical interventions, and only one live-attenuated JUNV vaccine, Candid#1, is available.^27^ This is partly due to challenges imposed by sequence variation exhibited across pathogenic GPCs (Figure S1), permitting few insights beyond the common goal of receptor blockade.^25,28^ While two distinct approaches of NWM GP1 targeting have been described,^25,26^ the structures and neutralization mechanisms of other therapeutically effective JUNV-targeting antibodies, such as the monoclonal J199,^29,30^ remain unknown.

With the goal of efficiently determining *in situ* structures of GPCs in their native and functional states and to inform the development of therapeutics against NWMs, we applied single-particle cryoelectron microscopy (cryo-EM) to image GPCs directly in PV membranes. This approach revealed the overall architecture of the JUNV GPC in the membrane, its post-translational modifications, the interfaces that maintain its 3-fold symmetry, and the interactions that stabilize its pre-fusion, SSP-bound state in the membrane. Structures of two distinct JUNV-neutralizing antibodies, CR1–28 and J199, bound to the JUNV GPC installed in PV membranes, further illustrated their distinct modes of GP1 engagement but shared mechanism of receptor blockade. These insights were supported by functional assays on the JUNV pseudotypes used for structural analysis, demonstrating the power of a shared platform for evaluating GPC structure and function.

## RESULTS

### A platform for *in situ* structure and functional analysis of Junin GPCs

To determine the *in situ* structures of JUNV GPCs displayed on the surface of PV membranes, we performed single-particle cryo-EM on the concentrated PV particles (Figures 1B–1D). Fully functional GPCs were heterologously expressed in human 293TT cells and installed on pseudotyped murine leukemia viruses (MLV) carrying a GFP reporter plasmid (Figures 1E, 1F, and S2–S4). These PVs acted as direct reporters on GPC function when exposed to naive hTfR1-expressing cells^31^ (Figures S2 and S3) and also as direct targets for imaging by cryo-EM after isolation and concentration from culture supernatants by ultracentrifugation (Figure S4). Using this approach, we visualized and pinpointed intact GPCs on PVs alone and bound to a GFP-fused Fab of the neutralizing antibody CR1–28 (CR1–28Fab)^26^ (Figures S4 and S5).

### *In situ* structure of the functional Junin GPC

Selection of CR1–28Fab-bound GPC yielded isolated particles that, when classified and averaged, showed membrane-installed GPC with consistent Fab-bound ectodomain features, transmembrane structure, and visible luminal domains (Figures 1G–1I and S6). Processing from these particles ultimately yielded a C3 symmetric map of the CR1–28Fab-bound, PV-membrane-installed GPC with an overall resolution of 3.8 Å (Figure S6; Table S1). Despite the clearly resolved GPC core, the inherent flexibility in the linker between the transmembrane helix of GP2 and its globular, zinc-binding luminal domain limited the observed resolution of the latter (Figure S6). Collectively, these features reflected a map of the pre-fusion state of a mature and functional JUNV GPC trimer, demonstrating a full complement of CR1–28Fabs, an ordered SSP-containing integral membrane structure, and intact luminal domains (Figures 1G–1I).

### Architecture of the Junin GPC ectodomain

**Figure 2.**
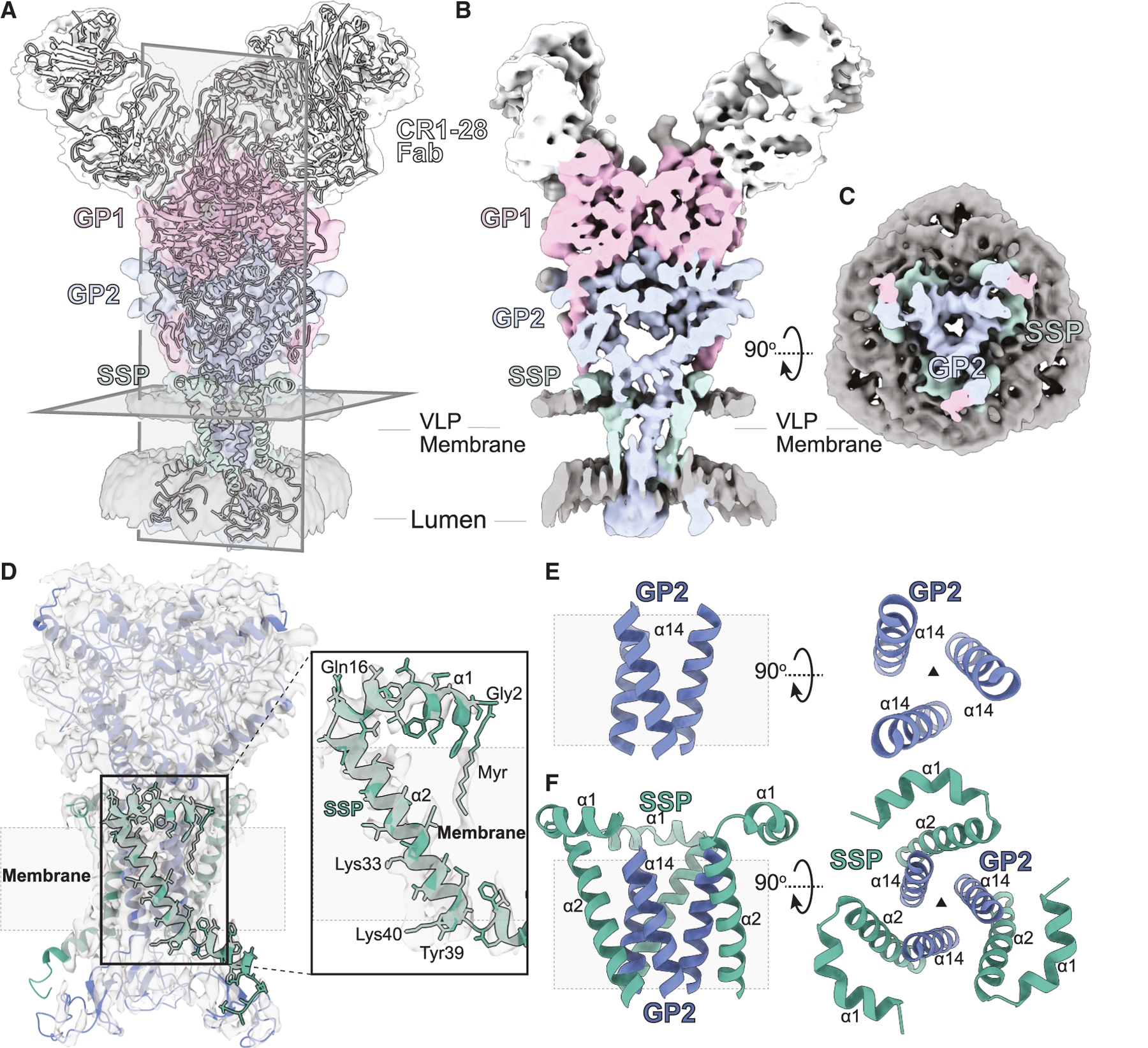
Structural features of the membrane-embedded GP2-SSP complex (A) Overlay of cryo-EM unsharpened density map (threshold = 0.3) and the CR1–28Fab JUNV GPC model with views perpendicular and along the 3-fold axis. (B and C) Central cross-section of the cryo-EM map (threshold = 0.3), with views perpendicular to (C) and along (B) the 3-fold axis. (D) View of the SSP and bound myristoyl group (Myr) in the cryo-EM density map (threshold = 1). (E) Side and top views of the GP2 transmembrane helices. (F) Side and top views of the GP2 and SSP transmembrane. The transmembrane region in side views is depicted as a light gray box with a dashed outline. See also Figures S6, S8–S13, and S15.

The 3-fold symmetric JUNV GPC ectodomain contains three sets of GP1, GP2, and membrane-anchored SSP (Figure S7). The topology and sequence register modeled for the SSP were informed by side-chain density fit and the placement of its N-terminal myristoyl group (Figures 2 and S8). The observed SSP sequence was confirmed through computational profiling of putative sequences onto its backbone. Each profile was modeled as anchored in a virtually reconstructed membrane, energy minimized, and scored by its fit into sharpened maps using Rosetta (Figure S8)^32^; this fold was not recapitulated by AlphaFold3 models of the complex (Figure S9), despite its overall similarity to membrane-extracted structures of known Lujo and Lassa GPCs (Figure S10).

A GP1 subunit caps the apex of the asymmetric unit of the JUNV GPC ectodomain (Figures S7A–S7C). The atomic model of GP1 residues 87–228 was informed by its known crystal structures.^25,26,33^ However, its N-terminal segment, spanning Ala61-His86, was built *de novo* (Figure S7B) and snakes along the surface of GP2, followed by a seven-stranded antiparallel β sheet (β2–β8) and five α-helical segments (α4–α8)^9,11^ (Figure S7C). The terminal segment of GP1, preceding the RxLx S1P cleavage motif, exits the core of the GPC at the interface between GP1 subunits atop GP2 (Figure S7C). Each GP2 principally associates with one GP1 and one SSP subunit (Figures S7A and S7D). An N-terminal segment of GP2, following its S1P cleavage motif, forms a β-hairpin, leading into its primarily helical (α9–α13) ectodomain. A single GP2 helix (α14) traverses the membrane, emerging into its zinc-binding luminal domain (Figure 2).

### Interfaces of the pre-fusion Junin GPC ectodomain

The stability of mammarenavirus GPCs and their 3-fold symmetry critically depend on both their non-covalent homomeric and heteromeric interfaces. Homomeric interfaces in the JUNV GPC collectively bury ∼13,835 Å^2^ of surface area, facilitated by a high degree of surface and charge complementarity (Figures 2A–2C and S11). GP1 helices α5-α6 and their adjoining loops form part of the inner core of the GP1 homotrimer. The terminal segment of GP1, following α8, exits the GPC core through an interface created by its own α4 and α6 and the α7 of its neighbor; it sits atop the loop bridging α11 and α12 of GP2 (Figure S7C). Likewise, homomeric interactions between GP2 subunits are stabilized by its N-terminal segment, which forms a loop nestled between the α9 helices of adjacent GP2 protomers (Figure S7D). GP2 helices are intertwined at the GPC core (Figure S7H) before each tethering their respective ectodomains to a transmembrane helix (Figure S7D).

A large heteromeric interface between GP1 and GP2 trimers stabilizes the JUNV GPC, burying 6,526 Å^2^ of surface area (Figure S11). Part of this interface is created by the N-terminal segment of GP1, which nestles against a hydrophobic cleft on GP2, contacting Trp362 and Trp395 on β9 and α13 (Figure S7B). In this cleft, it forms a strand, β1, that complements β9 in GP2 in an antiparallel configuration, facilitating polar contacts (Figure S7E). Exiting this cleft, hydrogen bond networks staple the adjacent GP1 and GP2 loops as GP1 nears the apex of GP2 and meets the most C-terminal residue visible for GP1, Arg247 (Figures S7F and S7G).

### Post-translational modifications of the Junin GPC

Post-translational modifications stabilize each domain of the JUNV GPC. Three glycosylation sites are observed on each GP1 domain, at Asn95, Asn166, and Asn178, with the N-linked glycan installed at Asn166 abutting the bound CR1–28Fab (Figure S12). Four additional glycosylation sites are observed at Asn357, Asn365, Asn382, and Asn387 on GP2 (Figure S12). These modifications remain highly conserved across other pathogenic NWM GPCs and are similar to those observed in the membrane-extracted recombinant structures of the Old World Lassa and Lujo GPCs (Figures S1 and S10).

A myristoyl group installed on the N-terminal Gly2 of the SSP is observed as a linear density protruding into the outer leaflet of the membrane bilayer (Figures 2D and S13). This anchors the SSP to the bilayer and dictates its topology in the membrane, matching that of the GP2 transmembrane helices (Figures 2D–2F). It also means that the SSP has anchor points on either side of the membrane bilayer, fixing the register and the angle of its transmembrane domain. This allows the SSP and GP2 helices, α2 and α14, respectively, to collectively form a 3-fold symmetric hetero-hexameric bundle in the membrane, with a group 1 topology (Figures 2E and 2F).

### Functional insights into the SSP register in the membrane and GP2-SSP interactions

The N-terminal helix (α1) of the SSP starts at Gly2 and remains parallel to the outer membrane surface, kinking twice: at Pro12 and after Gln16. The latter kink facilitates its insertion into the membrane at an ∼60° angle to its surface (Figure 2D). This geometry positions the hydrophobic patch between SSP Val24 and Tyr39 within the membrane (Figures 2D and F). Hydrogen bonds link GP2 serine residues Ser429 and Ser436 with adjacent carbonyls of SSP residues Ser27 and Gly34 (Figure S7I). Phe427, implicated in the attenuation of Candid#1 fusion activity upon its mutation to Ile^34^ (Figure S14), is stably buried within the transmembrane helix (α2) of the SSP. Lys33, also buried within the lipid bilayer, is partially stabilized by cation-pi interactions between Phe433 and Phe438 of neighboring GP2 protomers (Figures 2D and S13). In the luminal domain, the C-terminal SSP Cys57 is presumed to be disulfide linked to GP2 Cys469, creating a second anchor point for the SSP (Figure S13).

**Figure 3.**
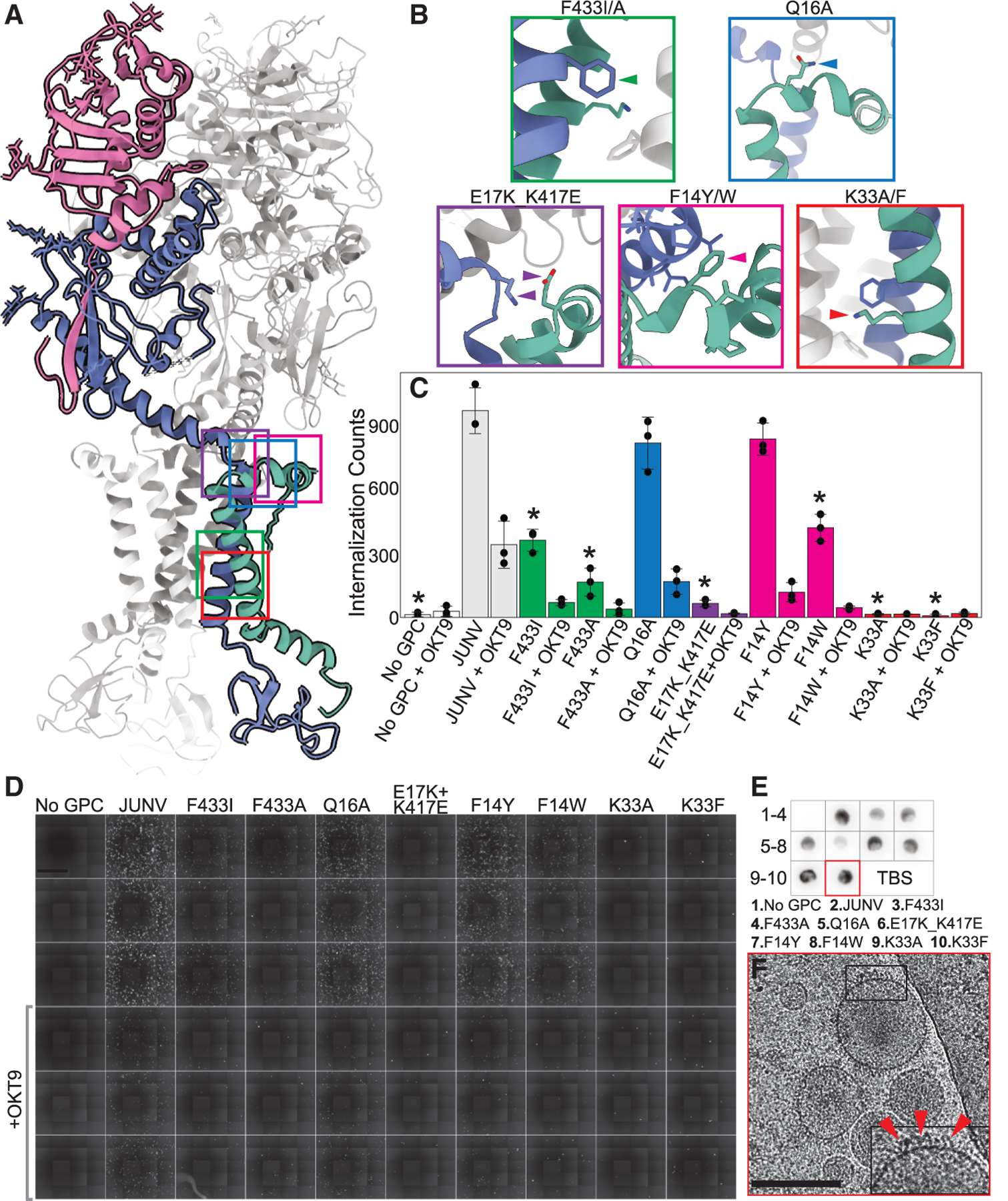
Mapping residue contacts important for JUNV GPC stability (A) Model of the JUNV GPC with key residues and their surroundings outlined by colored boxes. (B) Survey of highlighted boxes in (A), highlighting sequence alterations to the JUNV GPC; the box colors correlate to sites depicted in (A). (C) Quantification of GFP signal for mutant JUNV GPC PVs bearing the sequence variants indicated in (B) with 100 μg/mL OKT9. Data are represented as mean total GFP counts for each condition ± standard deviation. *p < 0.01, comparing uninhibited mutant GPC PV internalization relative to uninhibited JUNV PV internalization determined by Welch’s t test. *n* = 3 technical replicates for each condition. (D) Stitched montage images of GFP fluorescence in HEK293T cells depicting internalization of JUNV GPC sequence variants in (C). Scale bar, 1 mm. (E) Dot blot showing binding of CR1–28 against PV-containing media of sequence variants used for internalization assay in (D). (F) Cryo-EM micrograph of MLV particles pseudotyped with JUNV K33F GPC mutant; arrows point toward K33F GPC on PV surface. Scale bar 200 nm. See also Figure S14.

The importance of the observed SSP register in the membrane is highlighted by the functional impact of altering its predicted interactions with GP2. While mutations to non-interface residues (Gln16 and Phe14) had a mild effect on GPC function in PV internalization assays, altering interface residues of either GP2 or SSP, including Phe433, Glu17, Lys417, or Lys33, significantly disrupted internalization despite being detected on secreted PVs at near wild-type levels (Figure 3). In fact, PVs of the Lys33Phe variant appeared highly decorated in cryo-EM images despite its complete lack of internalization (Figure 3F). This indicated that the in-membrane cation-pi interaction exhibited by Lys33 better facilitates GPC function over the close-packed hydrophobic contacts expected from a Phe at that position.

### JUNV GPC targeting by CR1–28 and J199 antibodies

The CR1–28Fab-GP1 complex resembles that of its crystallographically determined structure,^13–15^ with a Cα root-mean-square deviation (RMSD) of 1.1 Å (Figure S15). The CR1–28Fab binds GP1 at its receptor binding interface, facilitated by interactions between a pocket on GP1 flanked by Asp113, Ile115, and Val117 and residue Tyr106 on the CR1–28Fab (Figure S15). This mimics the insertion of hTfR1 Tyr211 into the MACV GP1 receptor-binding site^25^ and is analogous to the interaction between the same GP1 pocket and residues Tyr30B and Tyr100A on neutralizing antibodies OD01 and JUN1, respectively^25^ (Figures S15A–S15D). In contrast, the full GPC structure does not permit the binding of GP1 to CR1–10, an antibody raised against recombinant GP1 that does not compete with CR1–28 and does not neutralize JUNV pseudotypes^26^ (Figures S15E and S15F).

**Figure 4.**
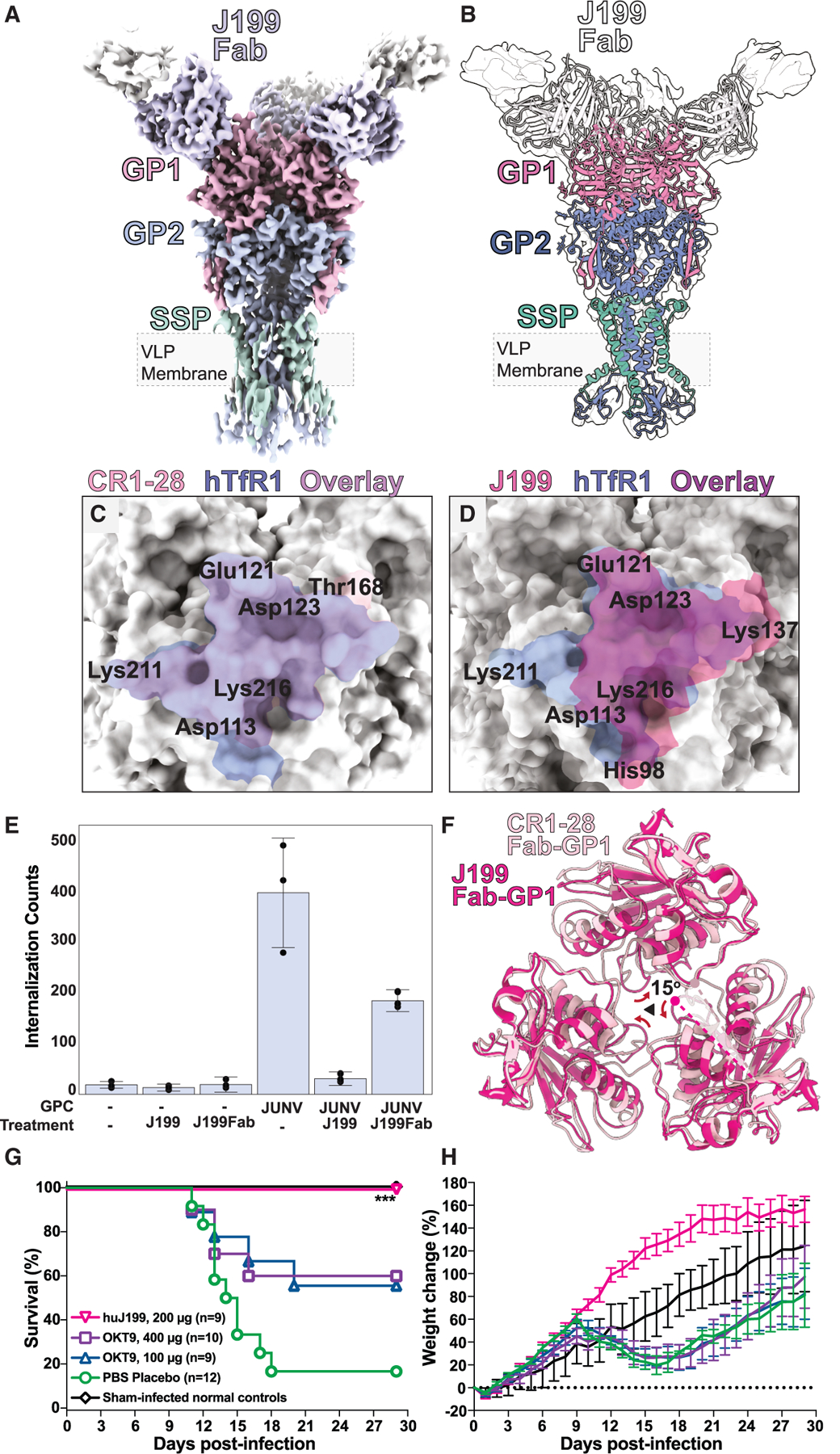
Structural insights into JUNV GPC inhibition by neutralizing antibody J199 (A) Cryo-EM map of J199Fab-bound JUNV GPC (threshold = 0.6). (B) Overlay of J199Fab-bound JUNV GPC model in unsharpened cryo-EM density map (threshold = 0.3). (C) Overlay of the hTfR1 (blue) and CR1–28 (light pink) footprints on the JUNV GP1. (D) Overlay of the hTfR1 (blue) and J199 (pink) footprints on the JUNV GP1. (E) Quantification of GFP signal indicating JUNV PV internalization into HEK293T cells in the presence of J199 or J199Fab. Data are represented as mean total GFP counts for each condition ± standard deviation. *n* = 3 technical replicates for each condition. (F) Overlay of J199Fab- and CR1–28Fab-bound JUNV GP1s; arrows depict a shift in domain architecture. (G and H) Survival (G) and body weight (H) of hTfR1 mice challenged with JUNV and treated intraperitoneally with either 400 or 100 μg OKT9, 200 μg huJ199, or PBS placebo 1 h pre- and 3 days post-infection. Animal weights, measured daily, represent the group mean and standard error of the percentage of change in the weight of surviving animals relative to their starting weights on the day of virus challenge. Sham-infected animals were included as normal controls. ^***^p < 0.001, comparing the huJ199 group to animals that received the PBS placebo. This *p* value is less than the Bonferroni-corrected threshold computed to adjust for multiple comparisons. See also Figures S5 and S16–S21 and Table S2.

Given the emerging evidence for distinct modes of JUNV neutralization by highly selective and JUNV-MACV cross-neutralizing antibodies, we chose to leverage our platform to study *in situ* JUNV GPC targeting by other, well-established neutralizing antibodies. We investigated the potent and therapeutically effective JUNV-selective antibody J199 (Figure 4),^29,30^ finding that it inhibited JUNV PV internalization into HEK293T cells to a similar or better degree than CR1–28 and better than saturating amounts of the hTfR1-neutralizing mouse monoclonal antibody OKT9 (Figure S16). Lacking a structural basis for J199 activity, we set out to determine its PV GPC-bound structure. J199Fabs were directly incubated with concentrated JUNV GPC pseudotypes for imaging and structure determination (Figure S17; Table S2). Following the same processing pipeline used to obtain the CR1–28Fab-GPC structure, we obtained a C3 symmetric map of the J199Fab-JUNV GPC at 3.8 Å resolution (Figures 4A, 4B, and S17; Table S2). That map showed sufficiently resolved density for the J199Fab to allow for the identification of its epitope and paratope; it revealed an interaction mediated in part by the insertion of Tyr105 into the GP1 cleft (Figure S18). The core of the J199Fab footprint on the JUNV GPC covers Glu121, Asp123, Lys137, and Lys216, several of which are shared with the CR1–28 footprint and also lie within the general footprint of hTfR1 (Figures 4C and 4D). A similar interaction is observed for lineage-specific Old World hemorrhagic fever-targeting monoclonals, such as the anti-Lassa 12.1F,^35^ whose footprint on Lassa GP1 is similar to those of J199 and CR1–28 on JUNV GP1 (Figures S19A, S19B, S19D, and S19E). This contrasts with the more broadly neutralizing anti-Lassa 25.10C,^36^ whose structure shows binding to an epitope at the GP1/GP2 interface (Figure S19).

### Insights into the potent JUNV GPC neutralization by J199

While GP1 residues in the J199 epitope display minimal structural rearrangement compared to their CR1–28-bound state, binding of the J199Fab induces a symmetric ∼15° rotation of GP1 trimers about the central 3-fold axis of the GPC (Figure 4F). That conformational change causes an overall compaction of the GP1 trimer interface that may lock what simulations indicate to be a dynamic pre-fusion state (Figures S20F and S20G), distinguishing GPCs bound by J199 from those bound by CR1–28, despite their similar modes of blockade.^25,26,37^

To assess the impact of J199-mediated pre-fusion JUNV GPC stabilization, we evaluated the ability of J199 and OKT9 to inhibit entry of PVs displaying Candid#1 GPC variants into human cells and also assessed the ability of J199 to inhibit replication of live Candid#1 in Vero cells. We found that 10 μg/mL of J199 fully inhibited the entry of Candid#1 GPC variants into human cells and blocked the replication of Candid#1 in Vero cells (Figure S21). We then compared the effectiveness of treating 20- to 22-day-old hTfR1-expressing mice challenged with the Romero strain of JUNV with a fully humanized version of J199 (huJ199), compared to OKT9 (Figures 4G and 4H), anticipating a difference as indicated by their differential blockade of JUNV-GPC-mediated internalization *in vitro*^28^ (Figure S16). The effect of treatment with huJ199 was compared to that of treatment with two different saturating concentrations of OKT9 administered 1 h before and 3 days post-infection (Table S3). While treatment with 200 μg of huJ199 protected 100% of JUNV-challenged animals, OKT9 dosed at either 400 or 100 μg only protected 60% and 56% of animals, respectively, while PBS placebo-treated mice had 17% survival (Figure 4G; Table S4). Animals treated with huJ199 showed clear weight gain and no signs of disease compared to OKT9- or PBS-treated mice, which experienced weight loss and recovery (Figure 4H).

## DISCUSSION

The pre-fusion structure of the JUNV GPC in a PV membrane serves as a platform on which to evaluate the architecture of pathogenic NWM GPCs, the impact of GPC sequence variants, and the effect of neutralizing agents. The *in situ* JUNV GPC structure is validated by the impact of sequence variants on the internalization of JUNV PVs into human cells (Figures 3 and S14), including chimeras harboring mixtures of JUNV GP1 or GP2 packaged with complementary components from the non-pathogenic Tacaribe virus (TCRV). While it is known and expected that radical changes to GPC identity reduce its abundance in cell lysates and particle-containing media and abrogate its ability to internalize into human cells (Figure S14), single point mutations introduced at GP2-SSP interface residues also substantially reduce GPC function (Figure 3), requiring structure-informed interpretation. This is likewise true of JUNV GPC residues altered to match those of the Candid#1 vaccine strain; their reduced internalization despite proper loading onto PV particles (Figures S14 and S21) is informed by structural insights.

The structure of JUNV GPC within a virus membrane also serves as a reference for investigating inhibitors of virus infection, whose activity may be influenced by the GPC-membrane interface.^38^ While the pre-fusion state of the JUNV GPC in PV membranes appears broadly similar to those of membrane-extracted NWM or Old World mammarenavirus GPCs (Figure S10), the nuances of the membrane-embedded GPC structure may differ from those in detergent-solubilized complexes or in mutationally stabilized structures and are not captured by AlphaFold3 models (Figure S9). This is evidenced by the atypical positioning of a membrane-ensconced Lys33 in α2 of the SSP (Figures 2, S8, and S13), which may partially destabilize the pre-fusion configuration of the GPC, facilitating its rearrangement during fusion. In fact, the GPC structure is sensitive to Lys33 mutation^39,40^; altering it to a hydrophobic amino acid abrogates the ability of the JUNV GPC to fuse (Figure 3). That effect is rescued by additional mutations of select residues in GP2,^40^ suggesting that residue networks at or near the membrane may be critical for facilitating fusion.

The structure of J199 adds new insights to the current models for direct JUNV GPC neutralization. To block receptor binding to GP1, JUNV-neutralizing antibodies tend to have epitopes that overlap with the hTfR1 footprint. J199 also binds within this footprint, engaging GP1 residues that are shared with the CR1–28 epitope and overlapping with the epitopes of neutralizing antibodies GD01, OD01, and JUN1, as well as the MACV-JUNV cross-neutralizing antibody CR1–07^25,26^ (Figures S15 and S18), which is known from crystallographic analysis. However, the *in situ* structure of J199Fab-GPC reveals an unexpected, cooperative reorganization of the GP1 homotrimer to a more closed state compared to the domain arrangement in the CR1–28-bound state (Figures 4, S11, and S20). While the functional consequences of this effect are not fully understood, this induced restructuring may play a role in the selective and potent inhibition of JUNV by huJ199, as demonstrated by its ability to protect against infection with lethal isolates Romero and Espindola in a cynomolgus monkey model of Argentine hemorrhagic fever (AHF)^30^ and its greater potency compared to blockade of hTfR1-mediated JUNV internalization alone (Figures 4G, 4H, S16, and S21).

### Limitations of the study

The insights we have gained from antibody-mediated neutralization of a heterologously expressed NWM GPC on PV membranes may not fully extend to unbound GPC in live infectious virus for several reasons, including GPC processing, installment of the SSP, and differences in GPC decoration on the virus surface. The poorly resolved features of the unbound JUNV GPC density further limit our ability to discriminate residue-level differences between its antibody-bound and unbound states (Figures S20A–S20E). Moreover, while our observed *in vitro* neutralization trends appear to be reflected *in vivo*, as evidenced by the potency of J199 against pathogenic JUNV in mice far exceeding that of the receptor-blocking antibody OKT9, this may occur because of J199’s interactions with the JUNV GPC but also potentially because it is humanized and distinctly interacts with the mouse immune system. Overall, heterologous expression systems, like the PVs described here, invite further development in the search for potent virus internalization blockers, including small-molecule pharmacophores and broadly neutralizing antibodies.^25,26,29,37,38^ Our results underscore the need for direct comparisons with *in situ* structures of live, pathogenic NWM GPCs.

## RESOURCE AVAILABILITY

### Lead contact

Any further information and requests for resources and reagents should be directed to and will be fulfilled by the lead contact, Jose A. Rodriguez (jrodriguez@mbi.ucla.edu).

### Materials availability

All bespoke reagents generated as part of this study will be made available from the lead contact with a completed materials transfer agreement.

### Data and code availability

- Coordinates and maps of the CR1–28Fab-bound JUNV GPC are available from PDB/EMDB entries PDB: 9MEW and EMDB: EMD-48221. Coordinates and maps of the J199Fab-bound JUNV GPC are available from PDB/EMDB entries PDB: 9N0D and EMDB: EMD-48781. The corresponding raw data for CR1–28Fab- and J199Fab-bound GPC are available from EMPIAR under entries EMPIAR-12718 (CR1–28) and EMPIAR-12719 (J199). Mass spectra are available from the MassIVE database under entry MSV000097881.
- This article does not report any original code.
- Any added information required to re-process the data in this article will be made available by the lead contact.

## STAR★METHODS

### EXPERIMENTAL MODEL AND STUDY PARTICIPANT DETAILS

#### Cells

HEK293T/293TT (human embryonic kidney epithelial) cells were grown in 4:1 OptiMEM to DMEM mix (Life Technologies; Thermo Fisher Scientific) supplemented with 1–2% fetal bovine serum (FBS, Life Technologies; Thermo Fisher Scientific) and 1% penicillin-streptomycin (100 U/ml penicillin and 100 μg/mL streptomycin) in a humidified incubator at 37°C with 5% CO_2_.

CHO-Neo, CHO-hTfR1, CHO-TfR2^42^ cells were grown in Ham’s F-12 Nutrient Mixture (F-12; Life Technologies; Thermo Fisher Scientific) supplemented with 10% fetal bovine serum (FBS, Life Technologies; Thermo Fisher Scientific) and 1% penicillin-streptomycin (100 U/ml penicillin and 100 μg/mL streptomycin) in a humidified incubator at 37°C with 5% CO_2_.

OKT9 hybridoma cells (ATCC-CRL-8021) were grown in Hybridoma Serum-Free medium (SFM) supplemented with 1% penicillin-streptomycin (100 U/ml penicillin and 100 μg/mL streptomycin) in a humidified incubator at 37°C with 5% CO_2_.

Vero cells (African green monkey kidney) cells were grown in 4:1 OptiMEM to DMEM mix (Life Technologies; Thermo Fisher Scientific) supplemented with 1–2% fetal bovine serum (FBS, Life Technologies; Thermo Fisher Scientific) and 1% penicillin-streptomycin (100 U/ml penicillin and 100 μg/mL streptomycin) in a humidified incubator at 37°C with 5% CO_2_.

#### Mice

The hTfR1 knock-in (human *TFRC* replacing the mouse *Tfrc*) mice on a hybrid C57BL/6 × 129/SvEv background have been previously described.^53^ Equal numbers of male and female 20 to 22-day-old hTfR1 mice were obtained from the breeding colony at Utah State University. The mice were housed in a GM500 Green Line IVC system (Tecniplast SpA, Italy) in individually ventilated cages and fed Harlan Lab Block and tap water *ad libitum*. Room air temperature in the biosafety level 3 enhanced laboratory dedicated to Junín virus (JUNV) work was 72 ± 4°F with 30–70% air humidity. The room had a 12–12 h dark/light cycle. Animal procedures used in this study complied with regulatory standards set by the USDA and were approved by the Utah State University Institutional Animal Care and Use Committee.

### METHOD DETAILS

#### Plasmids for pseudovirus production

JUNV GPC PVs were generated through recombinant expression of the JUNV GPC codon-optimized sequence from Junin virus strain MC2 within mammalian expression vector pcDNA 3.1+, an eGFP reporter plasmid in the pQCXIX retroviral vector, and MLV gag and pol genes, as previously described.^19,54^ For PV internalization assays, the eGFP construct was appended to an N-terminal SV40 nuclear localization signal. Full plasmid sequences were obtained for all constructs through Primordium sequencing. Sequence variants encoding JUNV GPC mutants were generated by cloning the JUNV GPC coding sequence with specified mutations into the JUNV GPC vector by Twist Bioscience (Tables S5–S7).

#### Pseudovirus production for internalization assays

293TT cells at 70–80% confluence in a 96 well plate were transfected with the MLV gag/pol, NLS-GFP, and GPC vectors using FuGene transfection reagent. 8 h post-transfection, the media in each well was replaced with fresh media. 60 h post-transfection, 10% of the PV-containing media was transferred from the expression plate to fresh cells at 50% confluence in a 96 well plate. After 16 h of incubation, the PV-containing media was replaced with fresh media supplemented with NucBlue live cell staining solution. 48 h post addition of PV-containing media, images of treated cells were collected at 20X magnification using an Evos M7000 wide-field fluorescence light microscope for both DAPI and GFP channels. The measured overlapping images covered 12–20% of the surface area of each well and were stitched together into montages by the Evos analysis software.

#### Large-scale production of pseudovirus particles for cryo-EM

To generate concentrated JUNV GPC PVs, 293TT cells in 10–20 T75s, 100–200 mL total volume, at 70% confluence were co-transfected in a 1.2∶1∶1 ratio with plasmids encoding the JUNV GPC, the MLV gag/pol genes, and a GFP reporter gene. 8 h post-transfection, cell media was replaced and supplemented with 20 μg/mL OKT9 to reduce reinternalization of particles. Cell supernatants were collected 72 h post-transfection, and cell debris were removed through centrifugation at 600xg for 5 min, followed by clarification at 2,500xg for 10 min and 10,000xg for 30 min. PVs were concentrated from the clarified supernatant via ultracentrifugation at 80,000xg over a 20–30% sucrose cushion. The supernatant was discarded, and PV pellets were resuspended in TBS pH 8.0 and concentrated by ultracentrifugation at 80,000xg for 2 h. The final PV pellets were resuspended in 50–100uL TBS pH 8.0 for use in subsequent biochemical and structural analyses.

#### Production of antibodies

##### CR1–28

Plasmids encoding the heavy and light chains of CR1–28^26^ antibody were provided by Jonathan Abraham. HEK293T cells were transfected with plasmids encoding CR1–28. 8 h post-transfection, cells were harvested via trypsinization and seeded in a hyperflask. One week post-transfection, cell culture media was collected, and cell debris were removed by centrifugation at 600xg for 5 min, followed by clarification at 10,000xg for 20 min. The antibody-containing media was filtered through a 0.45 μm vacuum filter. CR1–28 was purified using a protein G column against a gradient of Glycine buffer, pH 2; eluted fractions showed a single peak that contained the desired antibody (Figure S5). Purified fractions were dialyzed into TBS pH 8.0 and concentrated with a 10 kDa spin filter to a final concentration of approximately 2 mg/mL.

##### GFP-fused CR1–28Fab

The sequence encoding the CR1–28 fab heavy chain was synthesized with a C-terminal Baojin-GFP^55^ and His-tag by Twist Biosciences. HEK293T cells were transfected with plasmids encoding the CR1–28 light chain and CR1–28 heavy chain fab at a 1:1 ratio. 8 h post-transfection, cells were harvested via trypsinization and seeded in a hyperflask. One week post transfection, cell culture media was collected, and cell debris were removed by centrifugation at 600xg for 5 min, followed by clarification at 10,000xg for 20 min. The antibody-containing media was filtered through a 0.45 μm vacuum filter. Antibody fabs were purified using a Ni-NTA column against a gradient of high imidazole in TBS buffer. Purified fractions were dialyzed into TBS pH 8.0 and concentrated with a 10 kDa spin filter to a final concentration of approximately 2 mg/mL.

##### OKT9

Hybridoma cells expressing OKT9 were grown in 1 L roller bottles in hybridoma SFM for one week. Cell culture media was collected. Cell debris were removed via centrifugation at 600xg, and clarified via ultracentrifugation at 10,000xg for 20 min. Antibody-containing media was filtered via a 0.45 μm vacuum filter and purified using a protein G column against a gradient of Glycine buffer, pH 2. Purified fractions were collected, dialyzed into TBS pH 8.0, and concentrated using a 10 kDa spin filter to a final concentration of ∼5 mg/mL.

##### GB03-BE08 (J199) Fab

J199 (BEI resources) was assessed prior to digestion by SDS PAGE. 0.5mg J199 was Ficin digested at 37°C for 5 h to yield J199Fab and dialyzed into TBS pH 8 to a final concentration of ∼0.45 mg/mL, then assessed for proper cleavage by SDS PAGE.

#### cryo-EM grid preparation

2 μL of concentrated JUNV GPC-pseudotyped particles, alone or incubated with 100 μg/mL CR1–28Fab or J199Fab for 30 min were applied to glow-discharged (PELCO easiGLow - Ted Pella) standard and 2 nm ultrathin carbon-coated R1.2/1.3 Quantifoil grids and placed in a Vitrobot Mark IV (Thermo Fisher Scientific) at 10°C in ∼100% relative humidity. Standard quantifoil grids were blotted using a blot force of 0 and blot time of 6 s, carbon-coated grids were blotted using a blot force of −4 and blot time of 1 s and plunge frozen into liquid ethane. cryo-EM grids were screened using a TALOS F200C TEM (Thermo Fisher Scientific) operated at 200 KeV and equipped with a direct electron detector (Apollo – Direct Electron). cryo-EM grids with favorable ice conditions were clipped using a SubAngstrom clipping station and stored in liquid nitrogen for high-resolution imaging.

#### cryo-EM data acquisition

cryo-EM data acquisition of CR1–28Fab-bound JUNV GPC-pseudotyped viral particles was performed on the UCLA CNSI Titan Krios G1 operated at 300 KeV using a Gatan K3 Summit direct electron detector. Movies were collected in super resolution mode at 81,000× (calibrated pixel size of 1.114 Å per pixel), over a target defocus range of −1.0 to −2.5 μm and a total dose of 40 e^−^/A^2^. A total of 41,612 movies across 7 datasets were recorded with SerialEM software.^47^

cryo-EM data acquisition of J199Fab-bound JUNV GPC-pseudotyped viral particles was performed on the NCCAT Titan Krios operated at 300 KeV using a Gatan K3 Summit direct electron detector. Movies were collected in super resolution mode at 105,000× (calibrated pixel size of 0.8276 Å per pixel), over a target defocus range of −0.6 to −2.0 μm and a total dose of 40 e^−^/A^2^. A total of 39,837 movies across 2 datasets were recorded with Leginon software.^46^

cryo-EM data acquisition of the non-bound JUNV GPC-pseudotyped viral particles was performed on the BNL LBMS Titan Krios operated at 300 KeV using a Gatan K3 direct electron detector. Movies were collected in super resolution mode at 105,000× (calibrated pixel size of 0.825 Å per pixel), over a target defocus range of −1.5 to −2.5 μm and a total dose of 50 e^−^/A^2^. A total of 10,856 movies were recorded with EPU software (Thermo-Fisher Scientific).

#### cryo-EM data processing

After CTF estimation, micrographs were curated, and 39,237 (CR1–28Fab-bound) or 38,676 (J199Fab-bound) micrographs were retained for further analysis. An initial set of particles was manually picked and used to train the Topaz model. Then, particles were picked automatically using the trained model and the extracted particles were subjected to 2D classification, followed by ab initio reconstruction. This reconstruction served as the reference for heterogeneous 3D refinements (C1 symmetry). Next, 3D refinement was carried out using non-uniform refinement, enforcing C3 symmetry.

Subsequent steps included 3D classification without alignment to identify the best-resolved classes, which were subjected to additional local refinements using a mask that included the trimeric protein but excluded the membrane, as well as global and local CTF refinements. Particles were then locally motion-corrected in cryoSPARC and subjected to further rounds of local refinement, 3D classification, and CTF refinement. An overall resolution of 3.8 Å for both CR1–28 and J199-bound structures was achieved for the entire particle, based on the 0.143 FSC threshold criterion.

The unbound JUNV GPC was processed using a similar pipeline in cryoSPARC from 10,790 micrographs; following 2D and 3D classification and further processing with C3 symmetry enforced yielded a map from 122,594 particles.

#### Model building

An initial model of the JUNV-GPC-CR1–28Fab was generated by docking the JUNV GP1-CR1–28 (5W1K)^26^ into the LASV GPC (7PUY)^14^ threaded with JUNV GPC residues in Rosetta. Relaxation of the model in the absence of density was conducted in Rosetta. The initial JUNV GPC model was docked into the cryoEM density in Chimera, and further relaxed into the density using Rosetta. The final model was built in Coot, then iteratively refined in Phenix. The final model was evaluated for proper geometric properties in Coot and for fit to the density by Q-score.

SSP sequence assignment, topology and register were defined by first building a backbone model and then populating sidechains, informed by AlphaFold3 predictions, and homolog structures. To determine the final sequence register of the SSP transmembrane domain, potential sequences were threaded into the model backbone with respect to a fixed starting position (shift 0) and scored within a virtual membrane in Rosetta against both the EMready^49^-sharpened and cryoSPARC sharpened density. Five independent trials were conducted for each possible register shift of the sequence and scored and averaged either across all residues in the modeled SSP, the transmembrane region of the SSP only, or across all residues in the CR1–28Fab-GPC. A model based on the docked structure of the crystallographic structure of the CR1–28Fab was used to build and refine the GPC-bound CR1–28Fab. Density specific side chain and backbone refinements were then carried out in Coot and Phenix. An initial model of the J199Fab Cα trace was obtained by relaxing an idealized Fab structure (CR1–28) into the J199Fab density; this was updated, informed by a Rosetta density fast relaxed AlphaFold-generated backbone onto which residue side chains modeled where clear density was observed Coot, and refined in Phenix. Sequences for regions of the model that corresponded to ambiguous density in the cryoEM map were further cross-validated using the sequences of peptides observed by bottom-up mass spectrometry. Any residue in the J199Fab model that did not clearly fit the cryo-EM density, and that was not detected by bottom-up mass spectrometry was stubbed to alanine or deleted from the backbone trace.

#### Bottom-up mass spectrometry

For bottom-up mass spectrometry, proteins were reduced, alkylated and digested with trypsin for bottom up proteomics. Peptides were separated by nano-HPLC (Easy Nano; Thermo) on a 200 mm C18 column running an 80 min gradient. Column effluent was directed to the ion source of an orbitrap mass spectrometer (QE Plus; Thermo) running in positive ion data-dependent tandem mode.^56^

#### Dot blots

PDVF membrane was activated in methanol for 30 s, then incubated in TBS-T buffer for 2 min. Four microliters of PV-containing media or cell lysate was added to the membrane and incubated for 10 min. The membrane was blocked in 5% NFDM in TBS-T for 30 min, then incubated with a 1:1000 dilution of CR1–28 or J199 at 1–2 mg/mL for 2 h. Then membrane was washed in TBS-T for 30 min, then incubated with anti-human H + L-HRP (CR1–28) or anti-mouse H + L-HRP (J199) antibody for 2 h, and washed in TBS-T 3 × 30 min. The membrane was incubated in chemiluminescence substrate for 5 min then imaged using an Azure biosystems chemiluminescence imager.

#### Preparation of Romero strain of JUNV

The molecular clone of the Romero strain of JUNV was rescued in BHK-21 cells as previously described^41^ and provided by Slobodan Paessler (University of Texas Medical Branch). The stock used in the present study was prepared from a single passage in Vero cells. The virus stock was diluted in sterile minimal essential medium and the animals challenged by intraperitoneal injection of 0.2 mL containing approximately a 3.5 × 10^4^ fifty percent cell culture infectious dose (CCID_50_) based on back titration of the challenge inoculum.

#### Treatment of JUNV-challenged hTfR1 mice with huJ199 and OKT9

Mice were weighed the morning of the infection and assigned to treatment groups so that sex and age were evenly distributed among the groups. The average weight per treatment group across the entire experiment varied by 1.5 g or less. Animals in each group were treated with an intraperitoneal injection of OKT9, huJ199, or sterile PBS 1 h before JUNV infection, with a second treatment administered 3 days post-infection (Table S3). Sham-infected, untreated mice were included as normal controls. The animals were observed 29 days for morbidity and mortality, and daily body weights were recorded. As a result of the very young age mice required for this model, three mice died within just a few days of the infection, one mouse each in the 100 μg OKT9, huJ199, and the sham-infected normal control groups.

#### Molecular dynamics simulations

JUNV GPC models, without bound fabs or post-translational modifications, were subjected to 100ns molecular dynamics simulations using the GROMACS all-atom OPLS force field.^57^ Models sampled at 10ns intervals were aligned based on their GP1 subunits and R.M.S.D. values were computed across all GP1 domains relative to their respective input structure.

#### Preparation of Candid#1

Candid#1 obtained from BEI resources (NR-469) was serially passaged in Vero cells following the procedures outlined by York & Nunberg.^27^ Virus stocks obtained from the cell culture supernatant were directly used in infection assays.

#### Candid#1 titer assays

Candid#1 was serially diluted in OptiMEM and incubated with J199 and CR1–28 antibodies at 10 μg/mL for 30 min at room temperature. Vero cells at 70% confluence in a 12-well plate were incubated with the Candid#1/Candid#1-antibody mixtures for 1 h at 37C. Following the incubation period, Candid#1-containing media was removed and replaced with a 1:1 mixture of 4%-FBS containing optiMEM and 1% Seaplaque agarose. Cultures were incubated for 10 days at 37C with 5% CO_2_, then fixed in 5% glutaraldehyde for 1 h. After fixation, the agarose overlay was removed and the fixed cells were incubated with 0.1% crystal violet for 45 min, then washed in water. Plaques were visualized and imaged at 4x magnification using an Evos.

### QUANTIFICATION AND STATISTICAL ANALYSIS

#### Quantification of pseudovirus internalization

To quantify pseudovirus internalization, GFP fluorescence events corresponding to single cells in stitched Evos images were counted using a custom MATLAB script. Average internalization counts across replicate wells were computed for each condition along with their standard deviation and plotted in R. Welch’s T-tests were evaluated for each experimental condition tested.

#### Analysis of JUNV-challenged hTfR1 mice with huJ199 and OKT9

For overall statistical significance in the analysis of Kaplan-Meier survival plots, we used the Mantel-Cox log-rank test. All statistical evaluations were performed using Prism 9 (GraphPad Software). The Bonferroni method was applied to correct for multiple comparisons between the experimental treatment groups (Table S4).

## Supplementary Material

1

Supplemental information can be found online at https://doi.org/10.1016/j.celrep.2025.115971.

## Figures and Tables

**Table:** KEY RESOURCES TABLE

REAGENT or RESOURCE	SOURCE	IDENTIFIER
Antibodies		

Monoclonal Anti-Junin Virus, Clone GB03-BE08 (J199)	BEI Resources	NR-43227
J199Fab	This paper	N/A
huJ199	Zeitlin et al.^30^	N/A
CR1–28	Clark et al.^26^	N/A
CR1–28Fab	This paper	N/A
OKT9	This paper	N/A
Goat anti human IgG (L + H)-HRP	ThermoFisher	Cat# 31412; RRID:AB_2282650
Horse anti-mouse IgG HRP	Vector Laboratories	Cat# PI-2000–1; RRID:AB_2336177

Bacterial and virus strains		

JUNV Romero strain	Emonet et al.^41^	N/A
Candid#1	BEI Resources	NR-469

Chemicals, peptides, and recombinant proteins

Immobilized Ficin	ThermoFisher	44881

Critical commercial assays		

SuperSignal^™^ West Pico PLUS Chemiluminescent Substrate	ThermoFisher	34580

Deposited data		

CR1–28Fab-bound JUNV GPC structure	This paper	9MEW
J199Fab-bound JUNV GPC structure	This paper	9N0D
CR1–28Fab-bound JUNV GPC density map	This paper	EMD-48221
J199Fab-bound JUNV GPC density map	This paper	EMD-48781
CR1–28Fab-bound JUNV GPC cryoEM movies	This paper	EMPIAR-12718
J199Fab-bound JUNV GPC raw cryoEM movies	This paper	EMPIAR-12719
Mass spectrometry of GB03-BE08 preparation	This paper	MSV000097881

Experimental models: Cell lines		

239TT	ATCC	CRL-3467
HEK293T	ATCC	CRL-3216
OKT9-Hybridoma	ATCC	CRL-8021
VERO	ATCC	CCL-81
CHO-Neo	Kawabata et al.^42^	N/A
CHO-hTfR1	Kawabata et al.^42^	N/A
CHO-TfR2	Kawabata et al.^42^	N/A

Recombinant DNA		

MLV gag pol	Radoshitzky et al.^19^	Table S3
eGFP	Radoshitzky et al.^19^	Table S3
NLS-eGFP	This paper	Table S3
JUNV	Radoshitzky et al.^19^	Table S3
TCRV*	This paper	Table S3
TCRV GPC	This paper	Table S3
CR1–28 LC	Clark et al.^26^	Table S3
CR1–28 HC	Clark et al.^26^	Table S3
CR1–28HC Fab-GFP	This paper	Table S3
CANDID GPC	This paper	Table S3
JUNV GPC-based sequence variants	This paper	Table S3

Software and algorithms

MAFFT	Katoh et al.^43^	v7
PyRosetta	Chadhury et al.^44^	Ref. 2015
Cryosparc	Punjani et al.^45^	v4.4.1
Prism	GraphPad	v9
Leginon	Suloway et al.^46^	v3.7
SerialEM	Mastronarde et al.^47^	v4.1
EPU	ThermoFisher	v3.6
ChimeraX	Goddard et al.^48^	v1.9
EMReady	He et al.^49^	v.2.1
Coot	Emsley et al.^50^	v1.21
Phenix	Liebschner et al.^51^	v1.21
MATLAB	The MathWorks Inc.	v9.13.0 (R2022b)
AlphaFold	DeepMind	AlphaFold3
GROMACS	https://doi.org/10.5281/zenodo.15006631	2022.2
Topaz	Bepler et al.^52^	v0.2.4

## Highlights

- Single-particle cryo-EM reveals an *in situ* structure of the Junin virus GPC
- GPC structures reveal binding to neutralizing antibodies
- The structures offer new insights into potent Junin neutralization by antibody J199
