# Supplementary material for: In situ insights into antibody-mediated neutralization of a pre-fusion Junin virus glycoprotein complex
Source: Cell Rep. Author manuscript; Available in PMC 2025 Sep 20. (PMC12450071; doi:10.1016/j.celrep.2025.115971)
Supplement: 1 [file NIHMS2099525-supplement-1.pdf]

Cell Reports, Volume *44*

Supplemental information

In situ insights into antibody-mediated neutralization of a pre-fusion Junin virus glycoprotein complex

Lily J. Taylor, Michael R. Sawaya, Jonna B. Westover, Chenyi Wang, Frederick Jimenez, Aldo J. Muñoz, Julian Whitelegge, Brian B. Gowen, Gustavo F. Helguera, Roger Castells-Graells, and Jose A. Rodriguez

Supplementary Figures

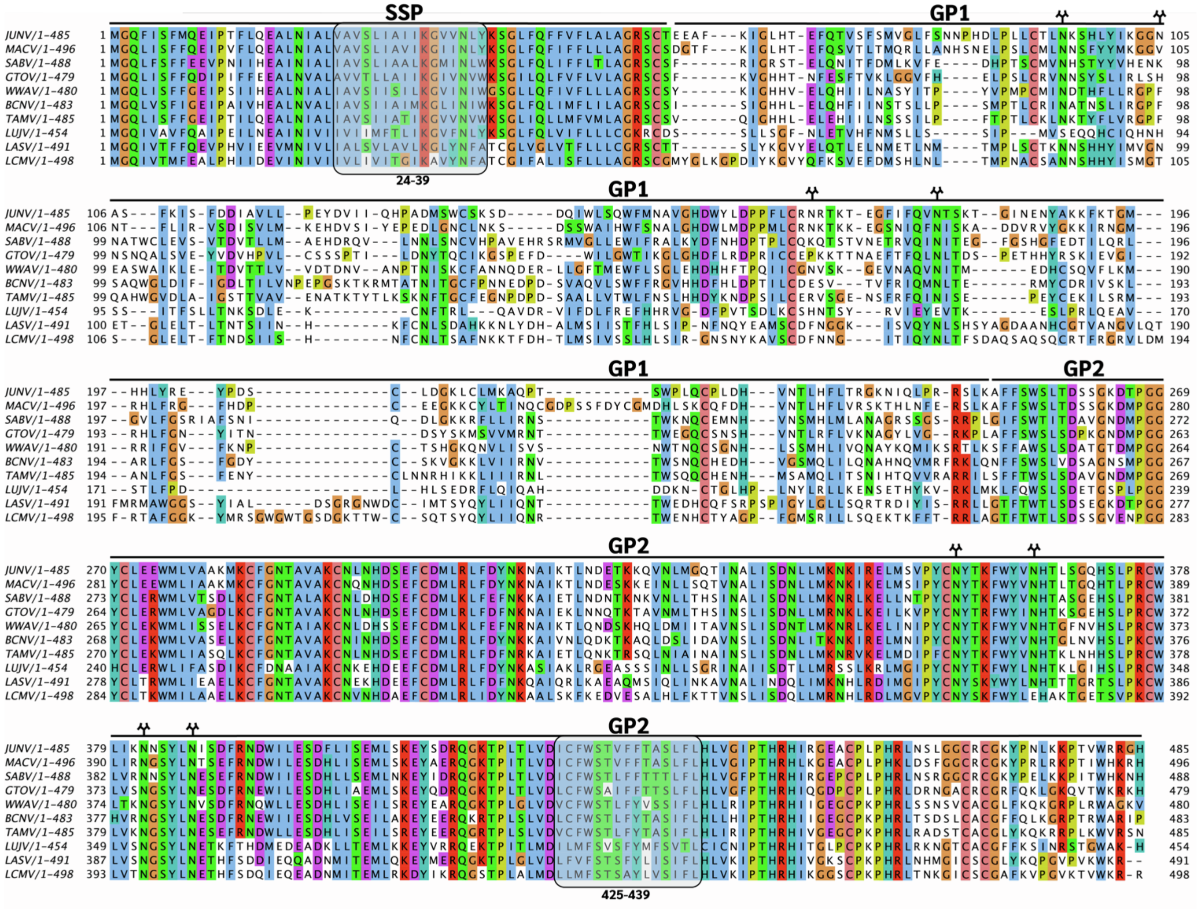

Figure S1. **Multiple Sequence Alignment (MSA) of select New World and Old World Arenaviruses, related to Figure 1**. Aligned GPC sequences from: Junin virus (JUNV) [P26313], Machupo virus (MACV) [Q6IUF7], Sabia virus (SABV) [Q90037], Guanarito virus (GTOV) [Q8AYW1], Whitewater Arroyo virus (WWAV) [Q911P0], Bear Canyon virus (BCNV) [A0PJ25], Tamiami virus (TAMV) [Q8AYY5], Lujo virus (LUJV) [C5ILC1], Lassa virus (LASV) [P08669] and Lymphocytic choriomeningitis virus (LCMV) [P09991]; for each, square brackets indicate its UniProtKB accession code. The SSP, GP1, and GP2 regions are indicated at the top, with N-linked glycosylation sites depicted as tree diagrams and transmembrane regions shown as rounded rectangles. MSA was performed using MAFFT [1] and Jalview [2]. Site-specific similarities between amino acids are highlighted with colored letters, following the Clustal color scheme (hydrophobic = blue, positive charge = red, negative charge = magenta, polar = green, cysteines = pink, glycine = orange, prolines = yellow, aromatic = cyan).

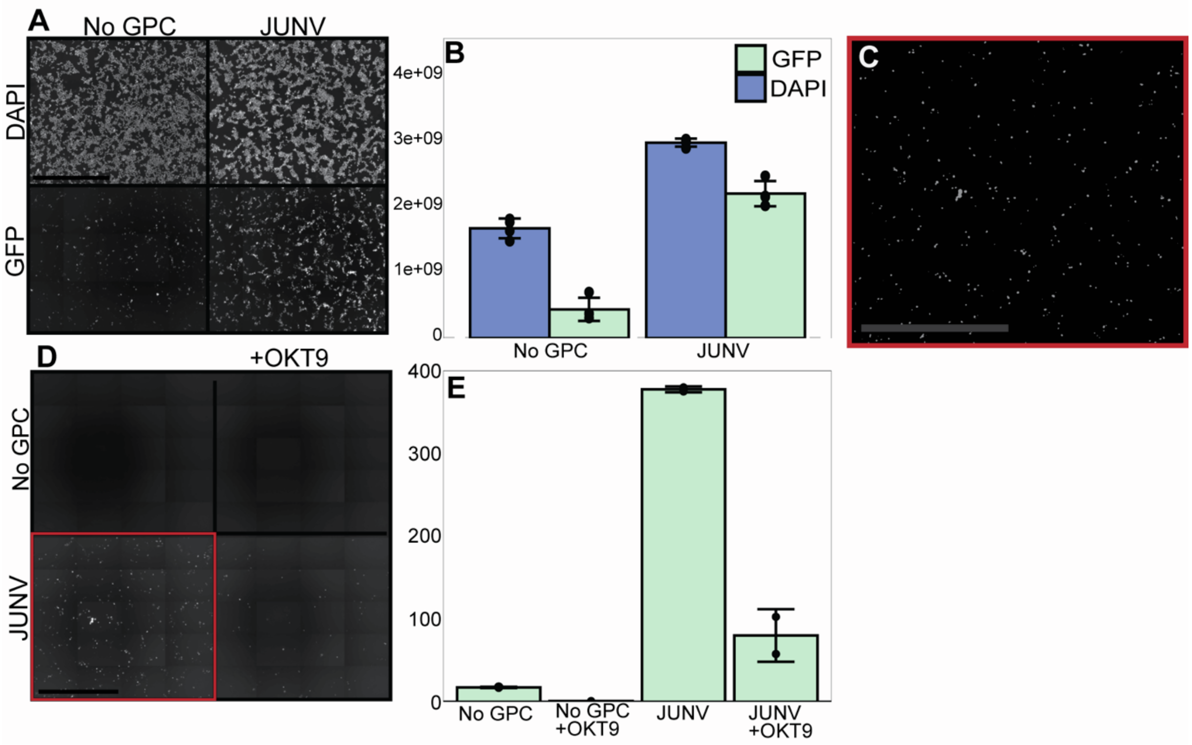

Figure S2. **Quantification of GPC-mediated internalization, related to Figure 1**. (A) Stitched montage images of DAPI and GFP fluorescence in 293TT cells expressing PVs used for internalization; scale bar, 500 µm. (B) Quantification of DAPI and GFP signal in (A); data is represented as mean total summed intensities for each condition +/- standard deviation, n=3 technical replicates. (C) Example internalization image of GFP channel showing GFP events used for quantification filled in grey; scale bar, 1 mm. (D) Stitched montage images of GFP fluorescence in HEK293T cells indicating internalization of JUNV GPC PVs, with OKT9 present at 100 µg/mL; scale bar, 1 mm. (E) Quantification of GFP signal in (D); data is represented as mean total GFP counts for each condition +/- standard deviation, n=2 technical replicates.

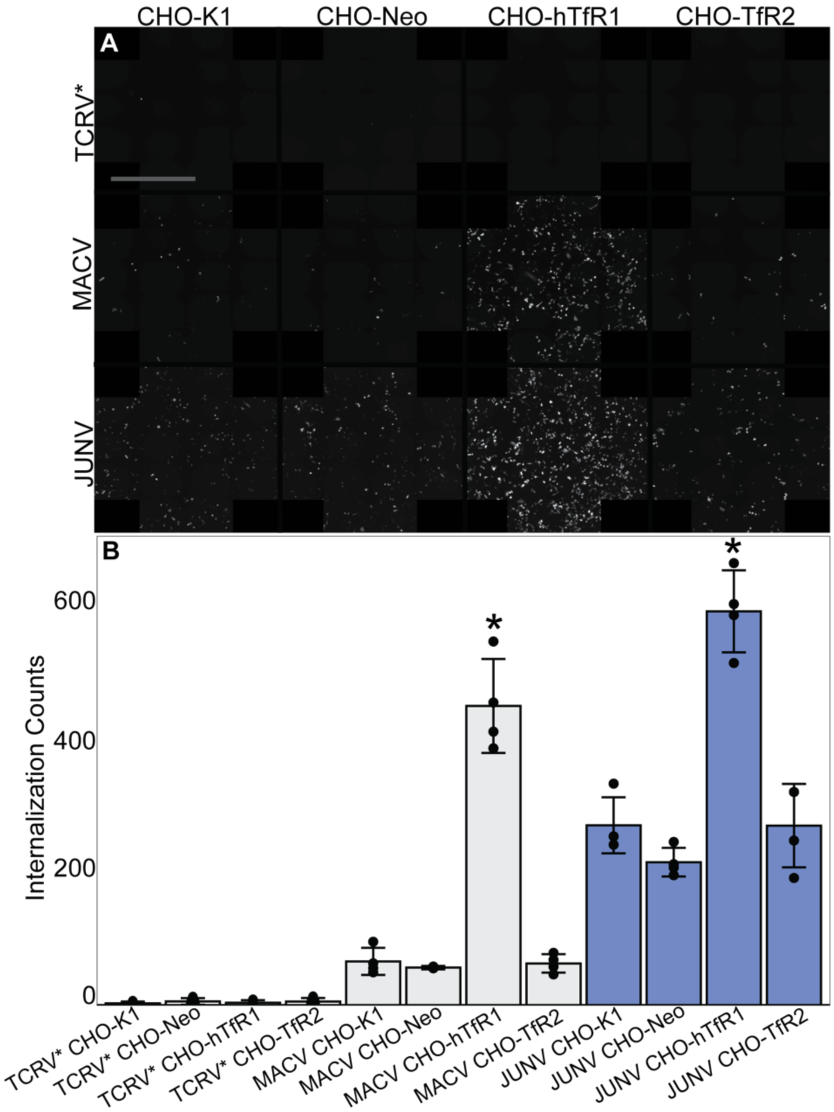

Figure S3. **Internalization specificity of GPC PVs, related to Figure 1**. (A) Stitched montage images of GFP fluorescence in CHO-K1 (endogenous transferrin receptor), Neo (no endogenous transferrin receptor), hTfR1 (no endogenous transferrin receptor, hTfR1-expressing), and TfR2 (no endogenous transferrin receptor, expressing hTfR2) cells indicating internalization of Machupo (MACV) and JUNV GPC PVs; scale bar, 1mm. (B) Quantification of GFP signal in (A); data is represented as mean total GFP counts for each condition +/- standard deviation. *p-value <0.01 comparing internalization of each pseudotype into CHO-Neo cell lines determined by Welch's T-test, n=4 technical replicates.

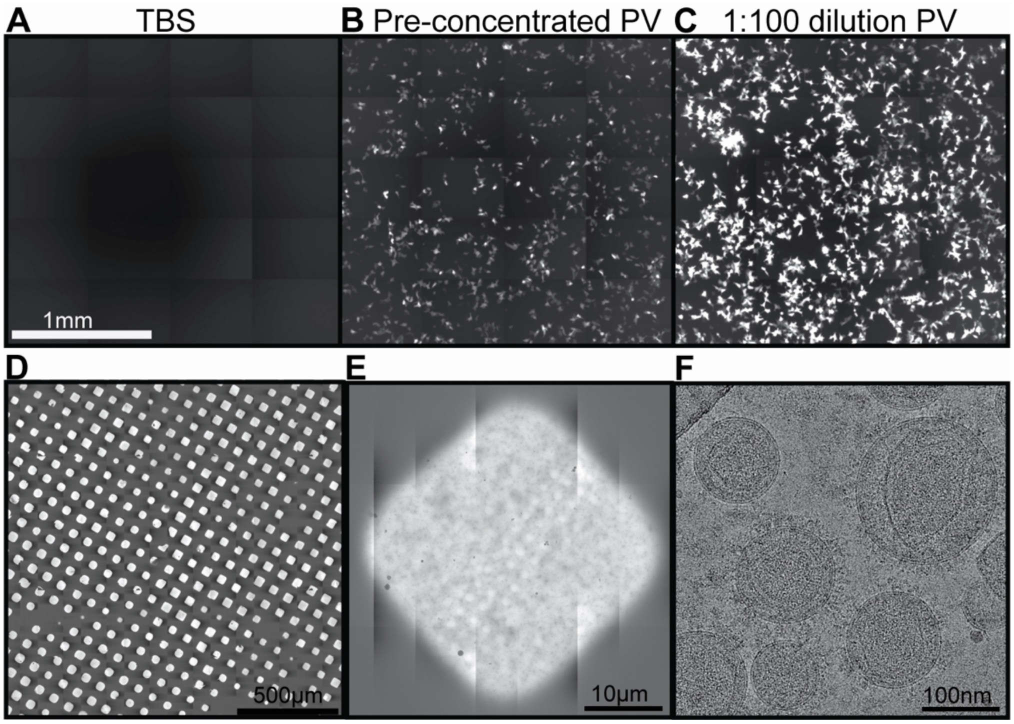

Figure S4. **Preparation of JUNV PV for cryo-EM imaging, related to Figure 1**. (A-C) Stitched montage images of GFP fluorescence in HEK293T cells depicting internalization of TBS control (A), pre-concentrated JUNV PVs (B) and concentrated JUNV PVs at a 1:100 dilution in TBS (C); scale bar, 1 mm. (D) Atlas of cryo-EM grid used for single-particle data collection; scale bar, 500 µm. (E) Example grid square used for single-particle data collection; scale bar, 10 µm. (F) Example hole used for single-particle data collection; scale bar, 100 nm.

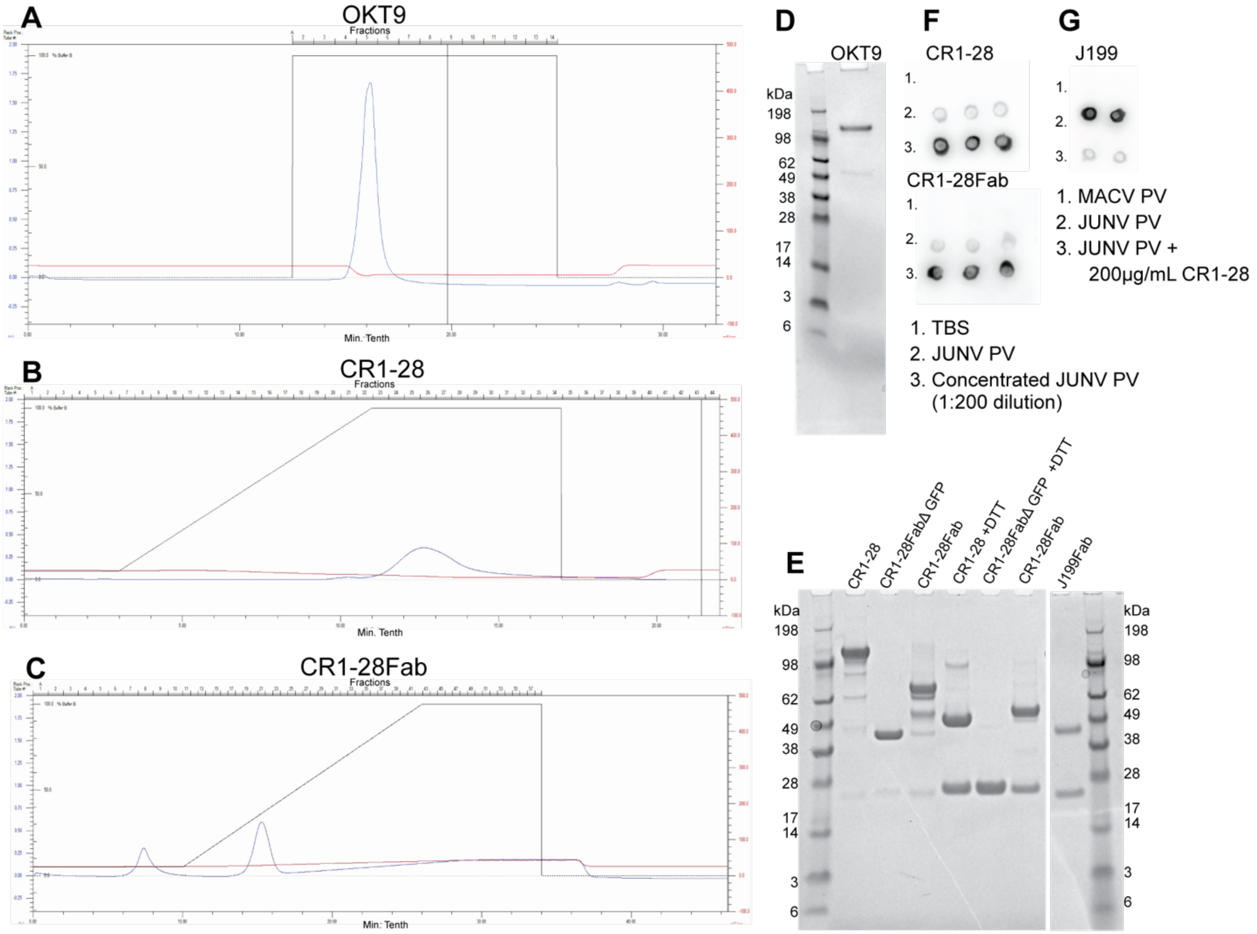

Figure S5. **Purification and validation of antibodies used in this study, related to Figures 1 and 4**. (A-B) Chromatogram from protein-G elution of purified OKT9 (A) and CR1-28 (B). (C) Chromatogram from Ni-NTA elution of CR1-28Fab. (D) SDS-PAGE gel of purified OKT9. (E) SDS-PAGE gel of purified CR1-28, CR1-28Fab, and CR1-28Fab without GFP fusion (CR1-28FabΔGFP) with 10 mM DTT and J199Fab. (F) Dot blots of 1) TBS, 2) JUNV PV, and 3) 1:200 dilution of concentrated JUNV PV in TBS against CR1-28 and CR1-28Fab. Anti-human IgG was used as secondary detection antibody. (G) Dot blot of 1) MACV PV, 2) JUNV PV, and 3) JUNV PV + 200 µg/mL CR1-28 against J199. Anti-mouse IgG was used as secondary detection antibody.

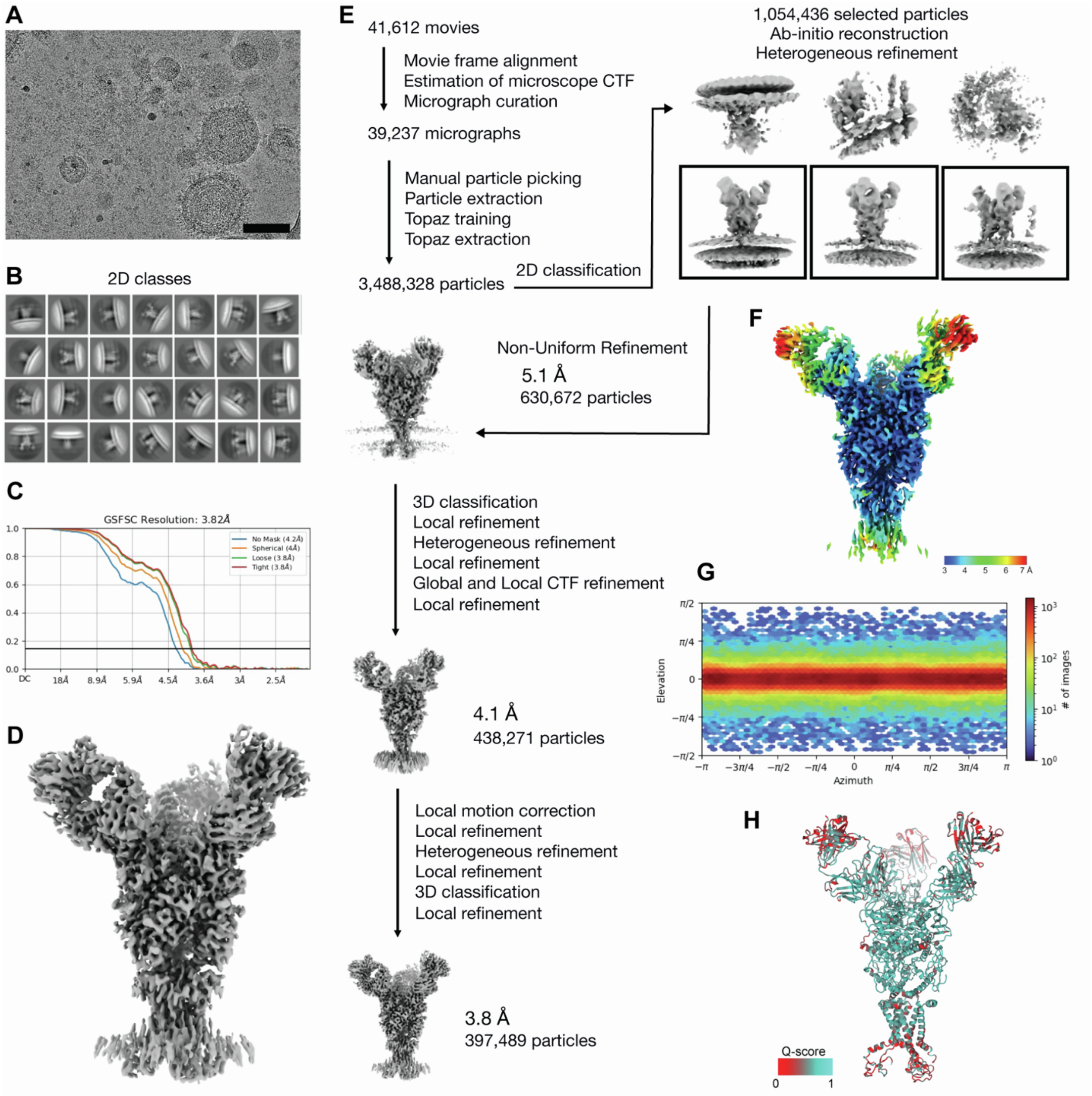

Figure S6. **cryo-EM data processing of the CR1-28Fab-bound JUNV GPC-pseudotyped virus particles dataset, related to Figures 1 and 2**. (A) Representative cryo-electron micrograph of CR1-28Fab-bound JUNV PV; scale bar: 100 nm. (B) 2D classes of the CR1-28Fab-bound JUNV GPC embedded in the viral membrane. (C) Gold-standard Fourier shell correlation curve with the 0.143 threshold indicated by a horizontal black line. (D) Sharpened final map at 3.8 Å. (E) cryo-EM data processing workflow. CTF = contrast transfer function. (F) Local resolution map for the JUNV GPC trimer. (G) The particle orientation density plot calculated using cryoSPARC is shown below the local resolution map. (H) CR1-28Fab-bound JUNV GPC model colored by Q-score values, which indicate the correlation between the built atomic model and the corresponding cryo-EM map[3].

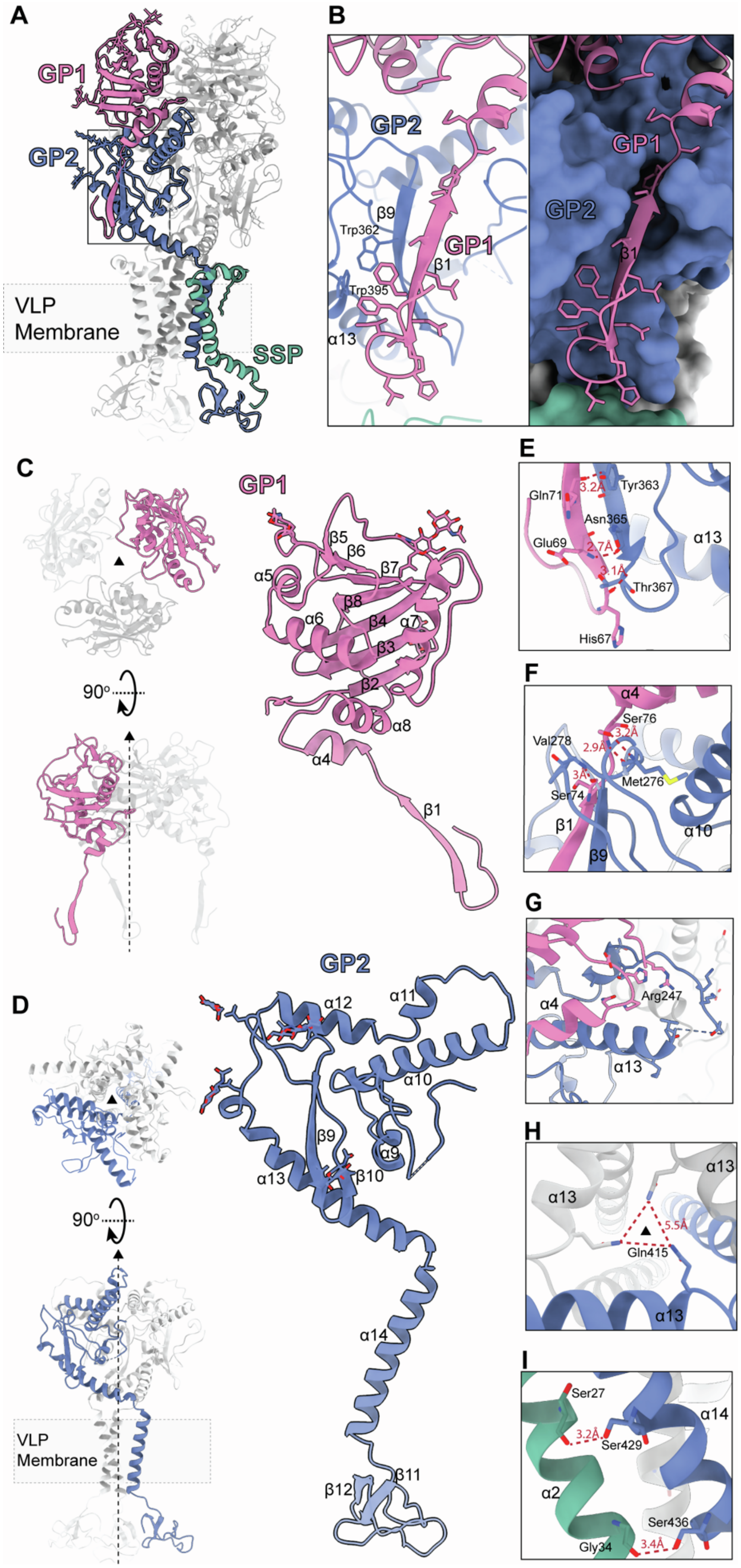

A
GP1
GP2
VLP
Membrane
SSP
B
GP2
β9
Trp362
GP1
β1
Trp395
α13
GP1
GP2
β1
C
GP1
β5
β6
α5
β7
β8
α6
β4
α7
β3
β2
α8
α4
β1
90°
D
GP2
α12
α11
α10
β9
α9
α13
β10
90°
α14
VLP
Membrane
β11
β12
E
Gln71
3.2Å
Tyr363
Asn365
α13
Glu69
2.7Å
3.1Å
Thr367
His67
F
α4
Ser76
3.2Å
Val278
2.9Å
3Å
Met276
Ser74
β1
α10
β9
G
α4
Arg247
α13
H
α13
α13
5.5Å
Gln415
α13
I
Ser27
α14
3.2Å
Ser429
α2
Ser436
3.4Å
Gly34

Figure S7. Architecture of the Junin GPC ectodomain and its stabilizing interfaces, related to Figure 1. (A) Asymmetric unit of the GPC, containing one of each: GP1 (pink), GP2 (blue), SSP (green); the PV membrane is indicated by a transparent gray bar. (B) View of the interaction between the GP1 N-terminus and GP2; side-by-side images show GP2 cleft in cartoon and surface forms. (C) Homotrimeric organization of GP1 subunits, with alpha helices (α4-8) and beta sheets (β1-8) as labeled. (D) Homotrimeric organization of GP2, with alpha helices (α9-14) and beta sheets (β9-12) as labeled. (E-G) Heteromeric contacts between GP1 and GP2. (H) View of homomeric interfaces in GP2. (I) Heteromeric contacts between GP2 and SSP with measured distances between neighboring residues indicated in red.

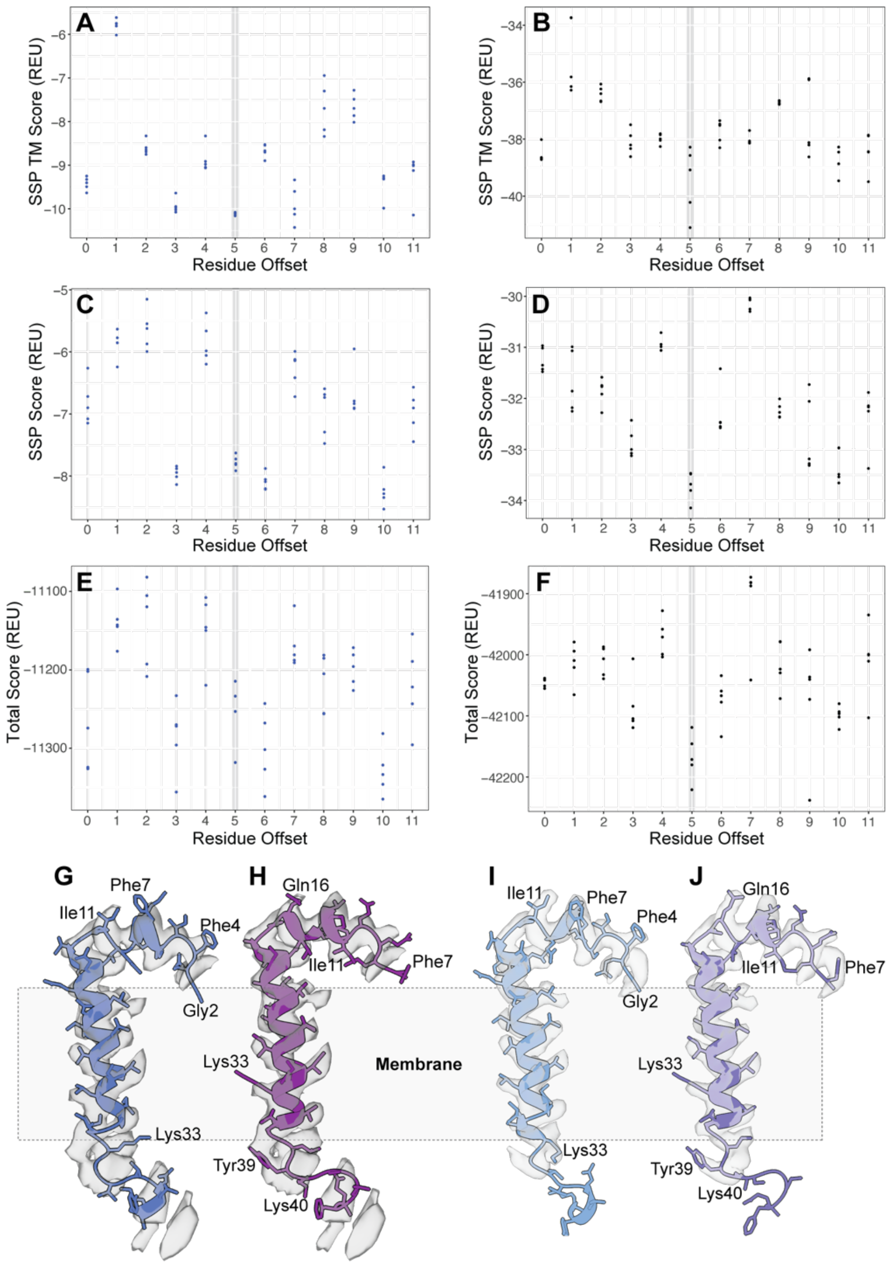

Figure S8. **3D profiling of SSP sequences, related to Figure 2**. Average scores are shown across SSP residues for each sequence shift variant across five trials, evaluated against the cryoSPARC sharpened map (A) or EMready map (B). Average scores across SSP transmembrane residues only, for each sequence shift variant across five trials, evaluated against the cryoSPARC sharpened map (C) or EMready map (D). Total complex score for each SSP sequence shift variant across five trials, evaluated against the cryoSPARC sharpened map (E) or EMready map (F). Gray bars indicate the overall chosen register. (G) Residues 2-40 of 0-shift density fast-relaxed JUNV GPC model in sharpened map at a threshold of 1. (H) Residues 2-40 of 5 residue-shift density fast-relaxed JUNV GPC model in sharpened map at a threshold of 1. (I) Residues 2-40 of 0-shift density fast-relaxed JUNV GPC model in EMready map at a threshold of 5. (J) Residues 2-40 of 5 residue-shift density fast-relaxed JUNV GPC model in EMready map at a threshold of 5.

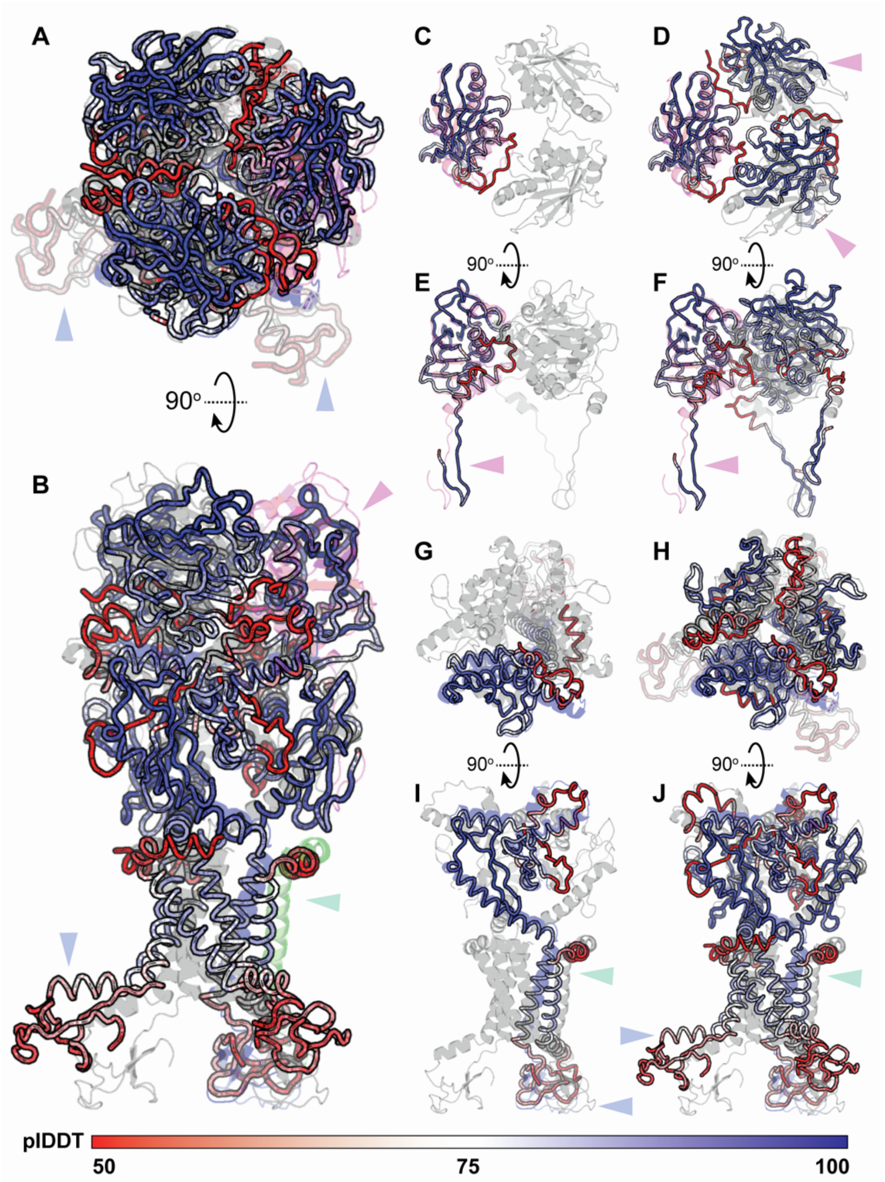

Figure S9. **Comparison of *in situ* JUNV GPC structure to AlphaFold3 (AF3) model, related to Figure 2**. (A-B) The JUNV GPC complex with a full complement of SSP, GP1 and GP2 subunits is shown as a translucent cartoon, with one asymmetric unit colored, SSP (green), GP1 (pink), GP2 (slate), the rest in gray. Aligned to and overlayed onto the GPC structure is the equivalent complex predicted by AF3, with its per-residue predicted local distance difference test (pLDDT) score colored from red (50) to blue (100). Views of the aligned AF3-predicted GP1 homotrimer are shown again overlayed onto the JUNV GP1 homotrimer structure from its top (C-D) and side (E-F). Views of the aligned AF3-predicted GP2 and SSP complex are shown also overlayed onto the JUNV GP2-SSP structure from its top (G-H) and side (I-J). Arrows point to regions of high incongruence between the JUNV GPC structure and the AF3 prediction.

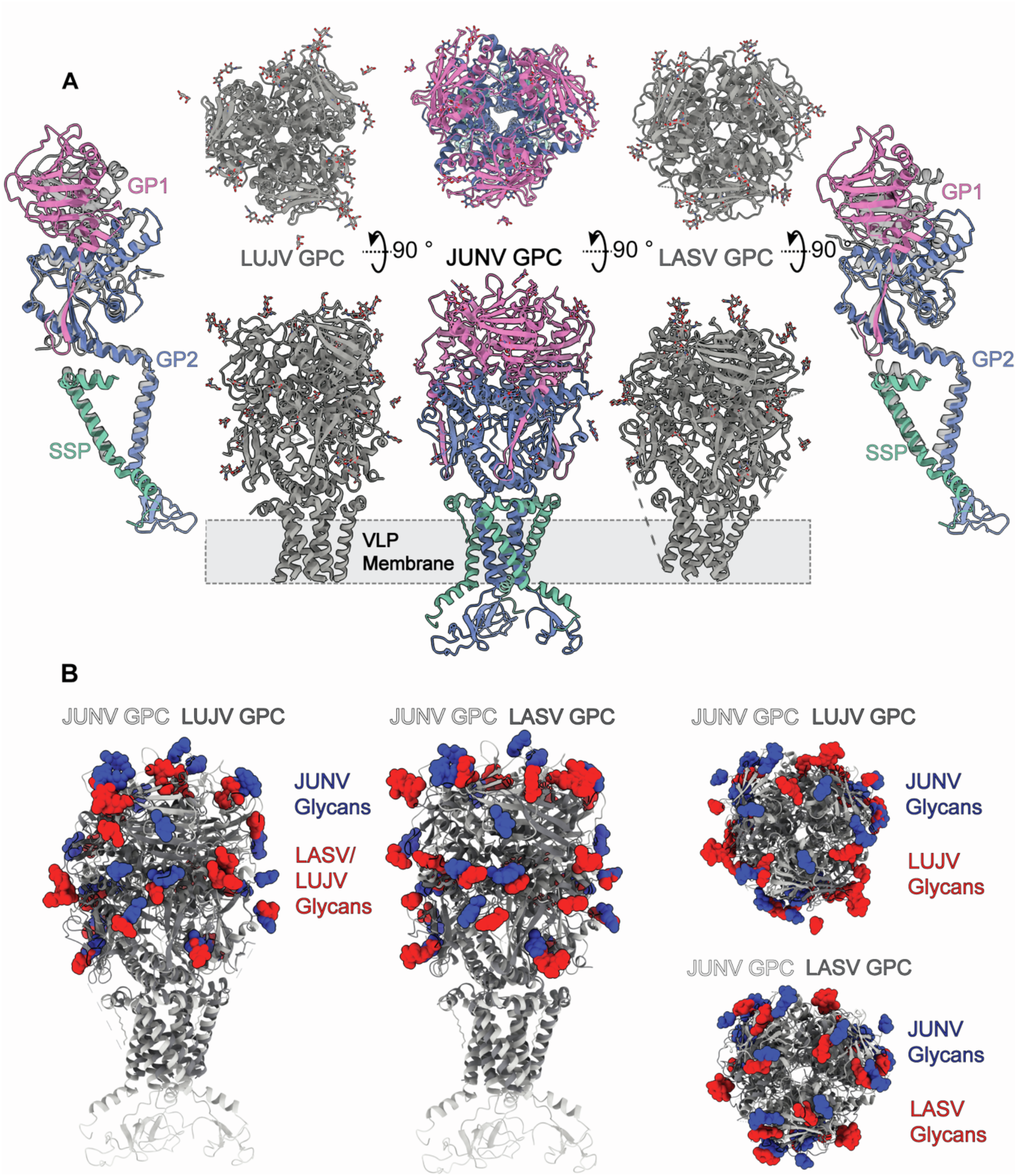

Figure S10. **Comparison of New World and Old World GPC structures determined by cryo-EM, related to Figure 2**. (A) View and alignment of Lujo (LUJV) (PDB: 8P4T) and Lassa (LASV) (PDB: 7PUY) GPC protomers to JUNV GPC model. Alignment was conducted for the GP2 helices of each protomer. (B) Alignment of LUJV and LASV GPC trimers to the JUNV GPC structure, with glycans shown as space-fill models; red: LUJV/LASV, blue: JUNV. Aligned models are viewed from the side (left) and top, looking down toward the membrane (right).

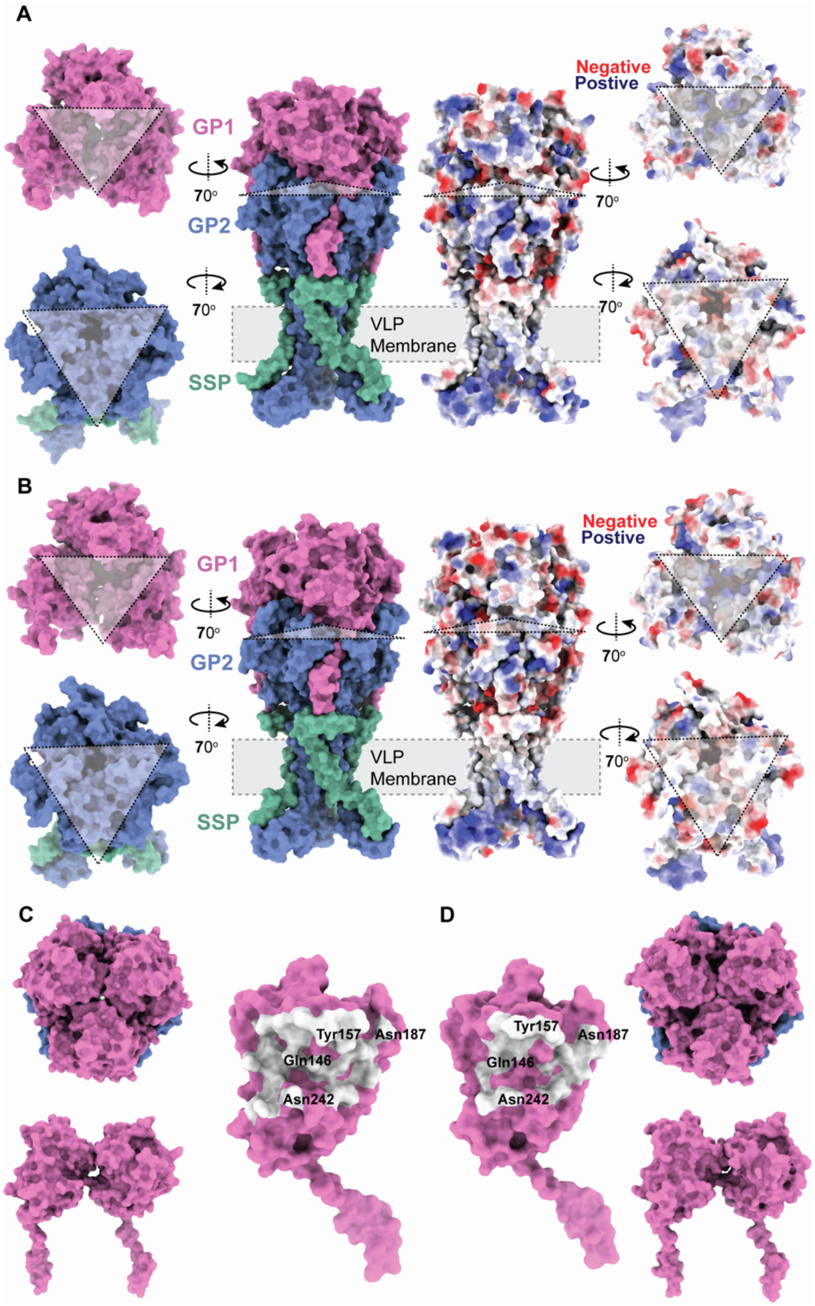

Figure S11. **Surface rendering of the CR1-28Fab-bound and J199Fab-bound JUNV GPC, related to Figure 2.** Surface rendering of the full-length CR1-28Fab-bound (A) and J199Fab-bound (B) JUNV GPC model colored by domain and electrostatic interactions. The full GPC complex is shown, as well as the interface between the GP1 and GP2 homotrimers. Positively charged regions are shown in blue, while negative regions are shown in red, and neutral regions shown in white. Triangular planes with dashed lines indicate the planar interface from which the GP1 and GP2 homotrimers were exfoliated and with respect to which they were tilted at a 70° angle. Surface rendering of the CR1-28Fab-bound (C) and J199Fab-bound (D) JUNV GPC model depicting a top view, two GP1 protomers, and the contact footprint of adjacent GP1 protomers shown in white.

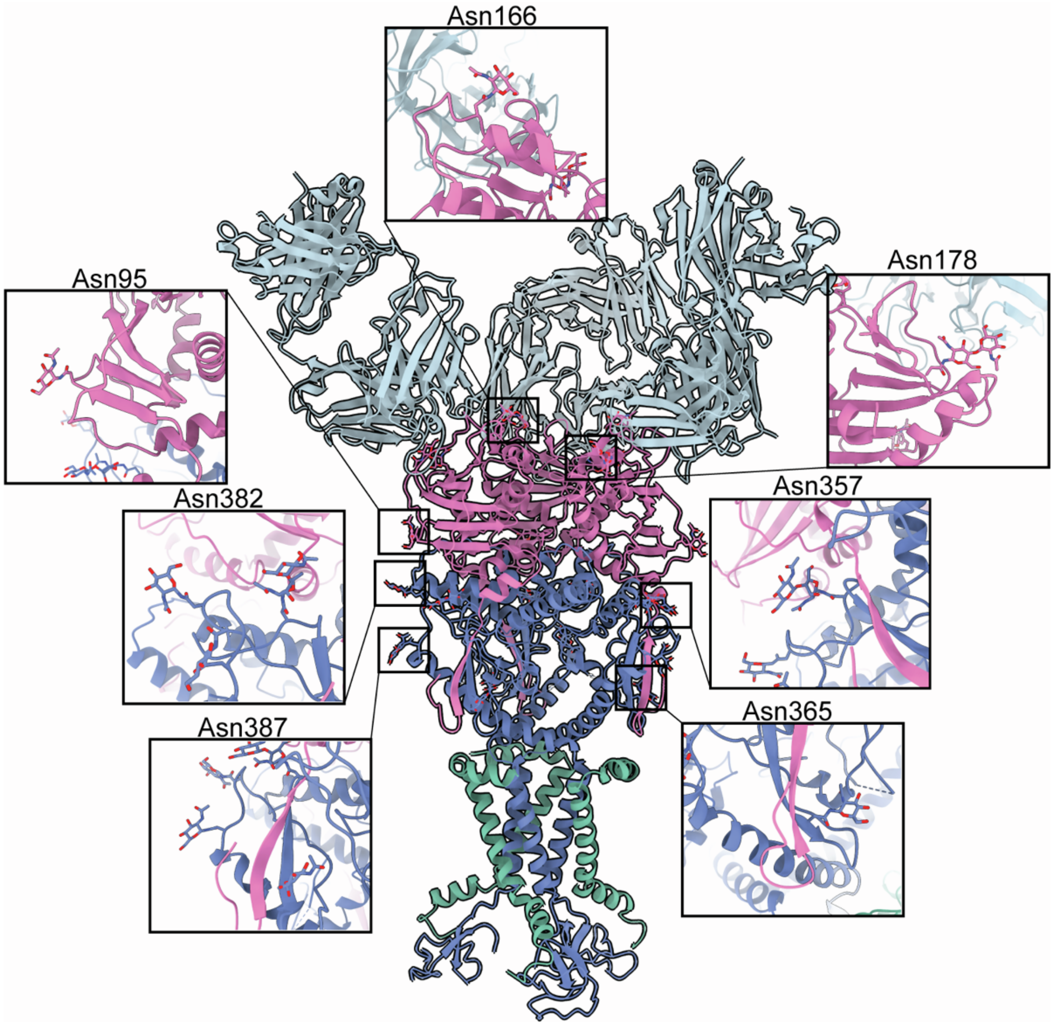

Figure S12. **Glycosylation sites across the JUNV GPC, related to Figure 2**. Each inset corresponds to an N-linked glycosylation site in the JUNV GPC. In total, three Asn-linked glycosylation sites are observed on GP1 (Asn95, Asn166, and Asn178), while four Asn-linked sites are observed on GP2 (Asn357, Asn365, Asn382 and Asn387).

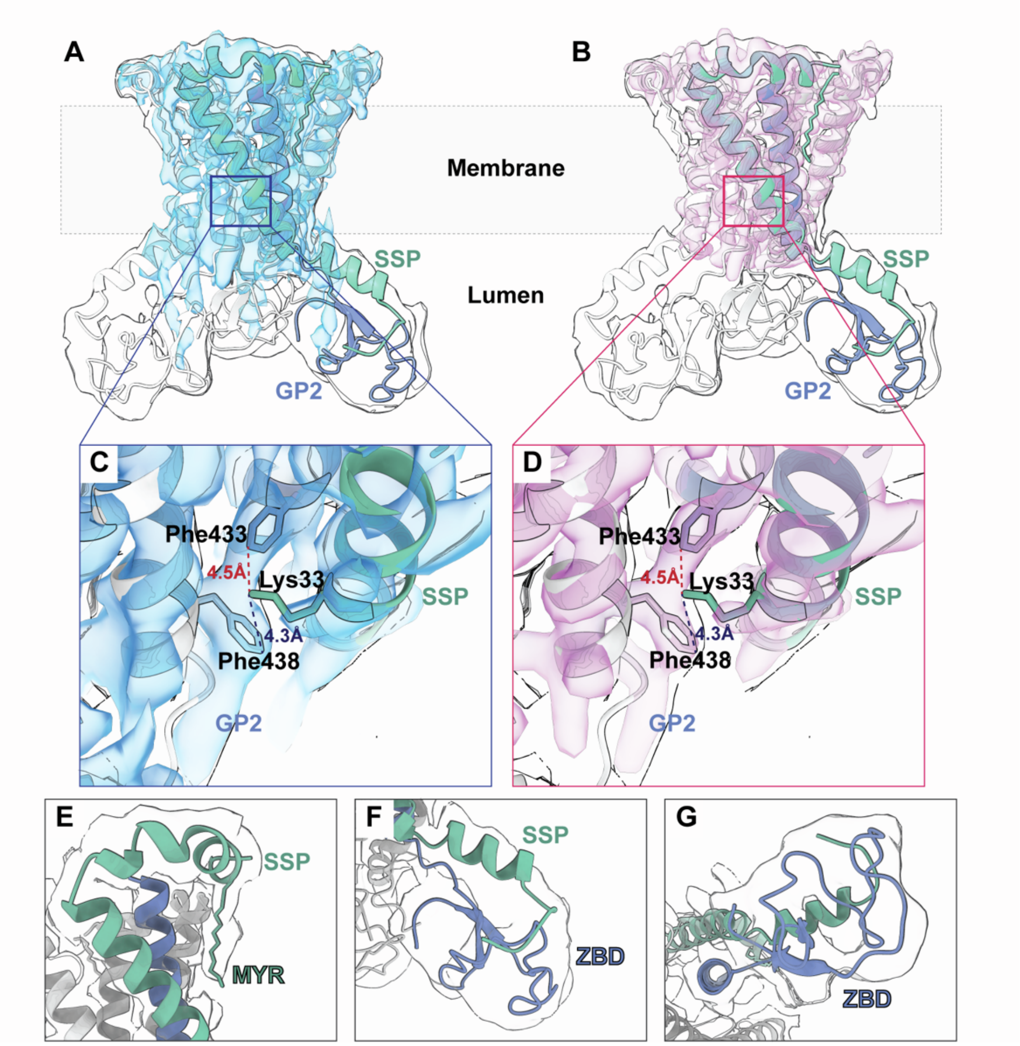

Figure S13. **Transmembrane and luminal structure of the GP2-SSP complex, related to Figure 2**. Side view of the GP2-SSP complex in the membrane, overlayed onto the unsharpened GPC map (grey) and either a cryoSPARC sharpened (A) or EMready processed (B) map. (C-D) Insets show regions of interest in the complex including the interaction of SSP Lys33 with GP2 Phe433 and Phe438 in the membrane, overlayed onto cryoSPARC sharpened (C) or EMready processed (D) maps. Additional insets show magnified views of the myristoyl group (MYR) installed at the N-terminus of the SSP (E), and orthogonal views of the luminal, zinc-binding domain (ZBD) of GP2 (F-G).

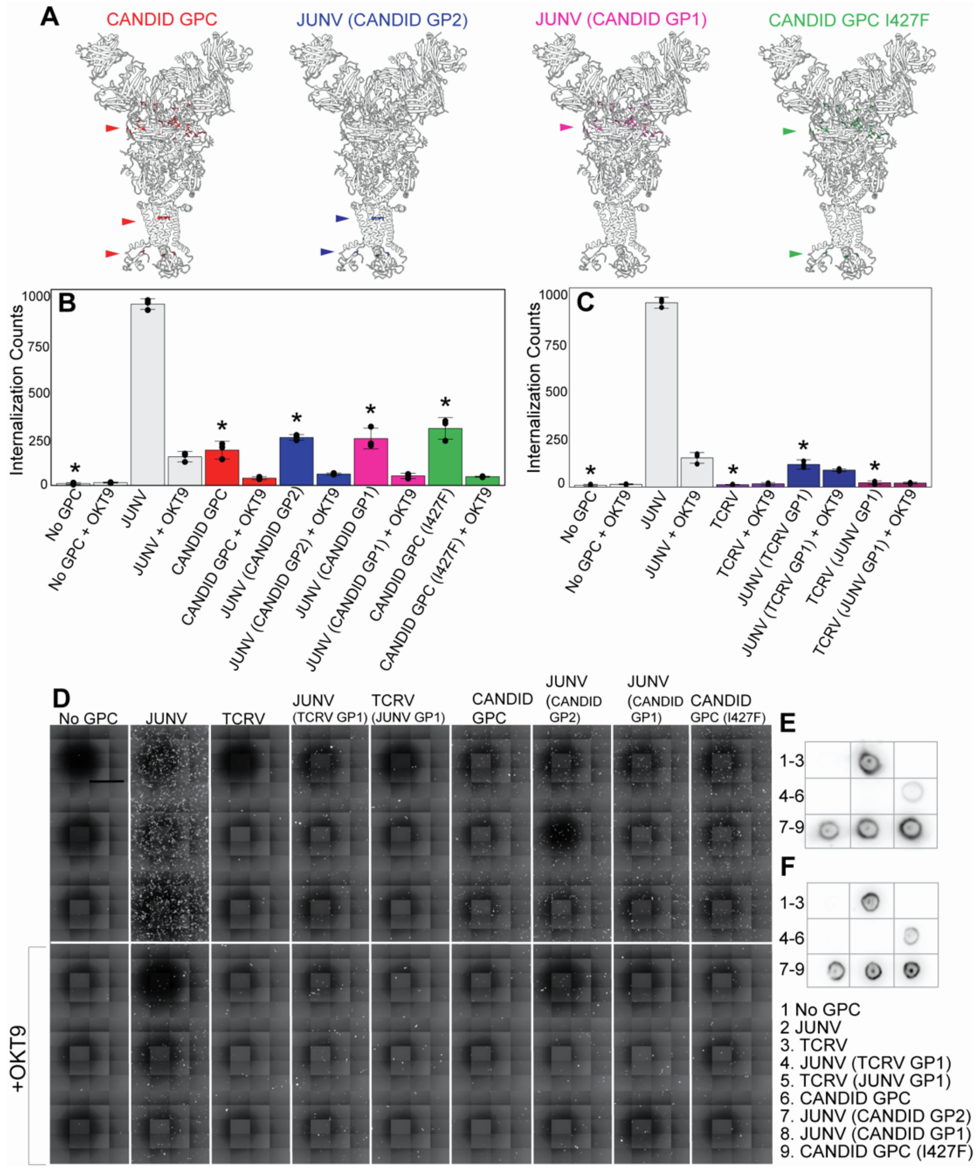

Figure S14. **Assessing the impact of JUNV mutations associated with the Candid#1 sequence, related to Figure 3**. (A) Models of the JUNV GPC with mutations from Candid#1 variants shown in color. (B) Quantification of GFP signal for mutant JUNV GPC PVs bearing the Candid#1 mutations indicated in (A) with 100 µg/mL OKT9. Data is represented as mean total GFP counts for each condition +/- standard deviation, n=3 technical replicates. *p-value <0.01 comparing uninhibited internalization of each sequence variant PV to JUNV PV determined by Welch's T-test. (C) Quantification of GFP signal for TCRV, JUNV, and TCRV/JUNV chimeras with 100 µg/mL OKT9. Data is represented as mean total GFP counts for each condition +/- standard deviation, n=3 technical replicates. *p-value <0.01 comparing uninhibited internalization of each sequence variant PV to JUNV PV determined by Welch's T-test. (D) Stitched montage of GFP fluorescence in HEK293T cells depicting internalization of JUNV GPC sequence variants in (C-D) with 100 µg/mL OKT9; scale bar, 1mm. (E-F) Dot blots showing chemiluminescence signal for binding of primary antibody CR1-28 against PV media (E) or cell lysates (F) after expression of variants in (C).

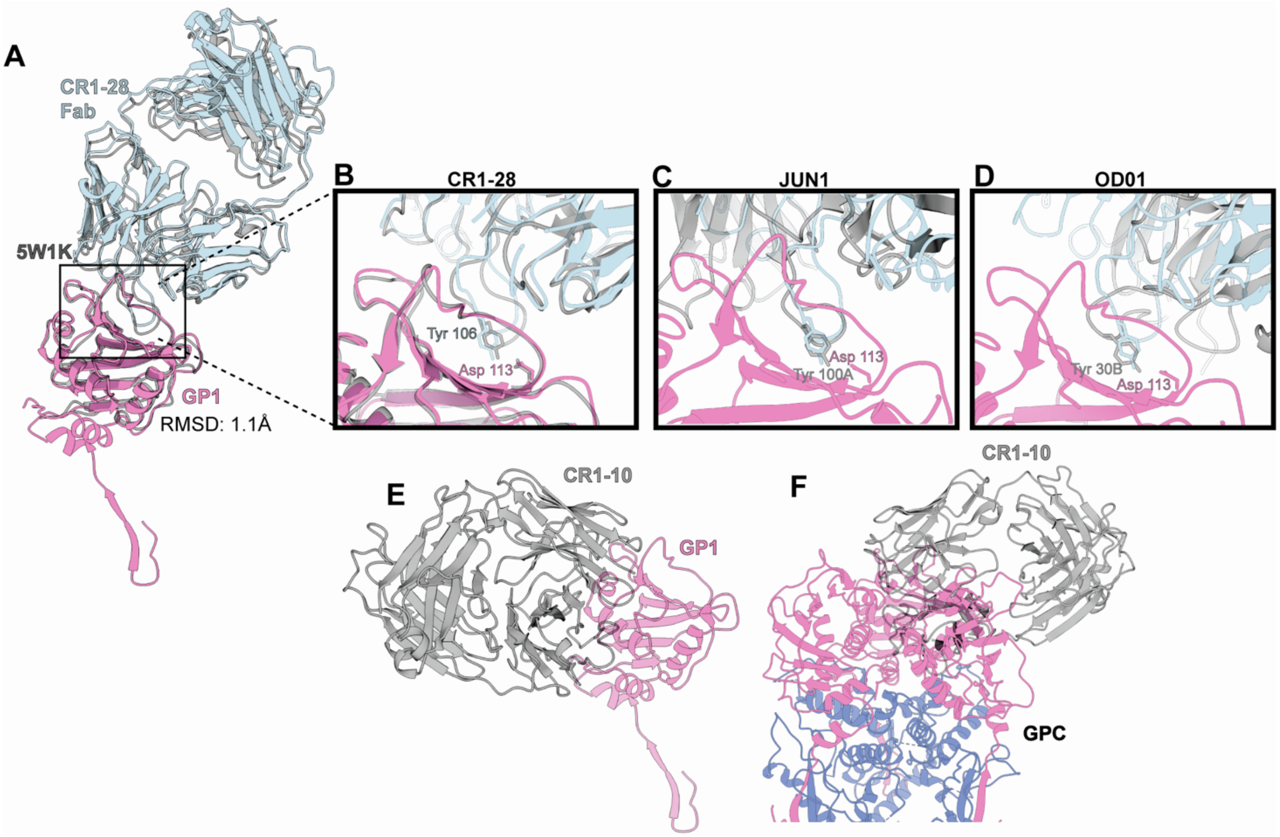

Figure S15. **Comparison of JUNV GP1-Fab interfaces, related to Figures 1 and 2**. (A) Overlay of GP1-CR1-28Fab with previously determined crystal structure, PDB: 5W1K. (B) View of GP1-CR1-28Fab interface. cryo-EM structure is shown in blue/pink, crystallographic structure in grey. (C) GP1-alignment of JUN1 antibody showing predicted interface. (D) GP1-alignment of OD01 antibody showing predicted interface. (E) GP1-alignment of CR1-10 antibody. (F) GP1-alignment of CR1-10 antibody in context of full GPC.

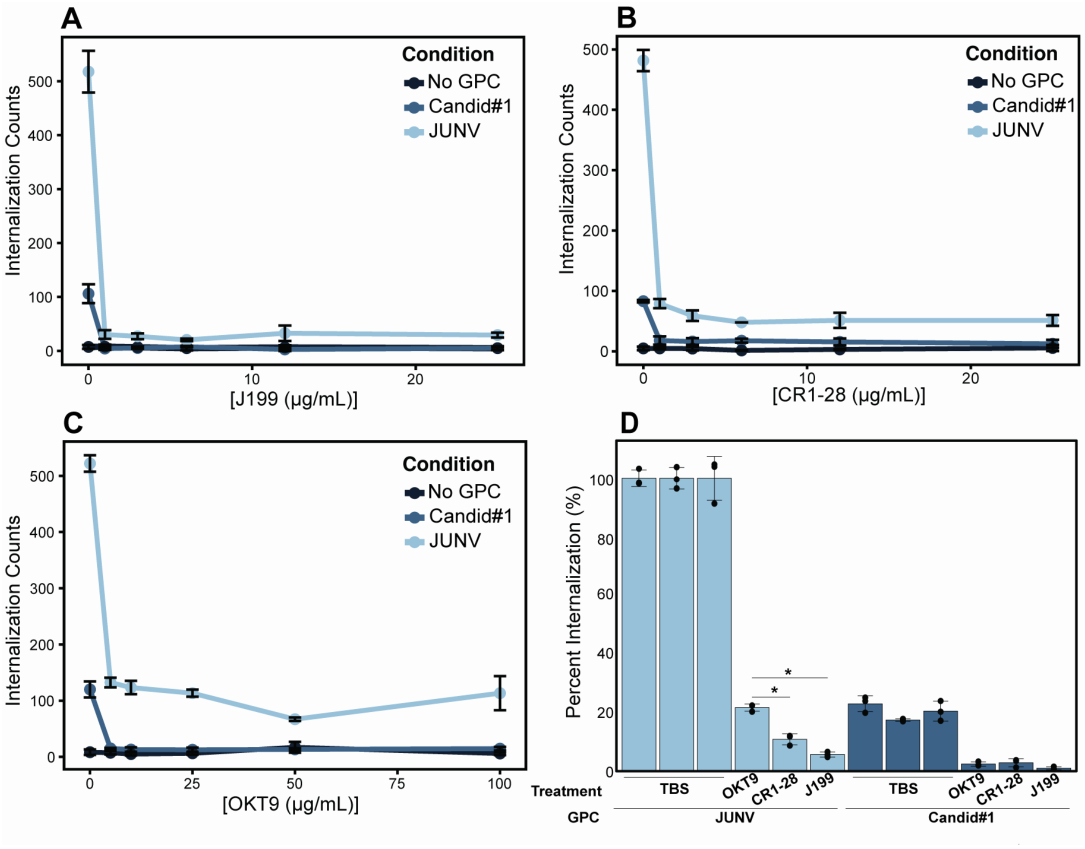

Figure S16. **Antibody-based neutralization of PV *in vitro*, related to Figure 4**. Internalization of no GPC, Candid#1 GPC, and JUNV GPC pseudotypes in HEK293T cells in the presence of various concentrations of J199 antibody (A) or CR1-28 antibody (B) or OKT9 antibody (C). Data is represented as mean total GFP counts for each condition +/- standard deviation. (D) Percent internalization of JUNV and Candid#1 GPC pseudotypes in HEK293T cells in the presence of no antibody (TBS) or 25 μg/mL of J199, CR1-28, and OKT9 antibodies relative to uninhibited JUNV pseudotypes. Data is represented as mean percent internalization for each condition +/- standard deviation. *p-value <0.01 comparing antibody-inhibited internalization of JUNV PV determined by Welch's T-test, n=3 technical replicates.

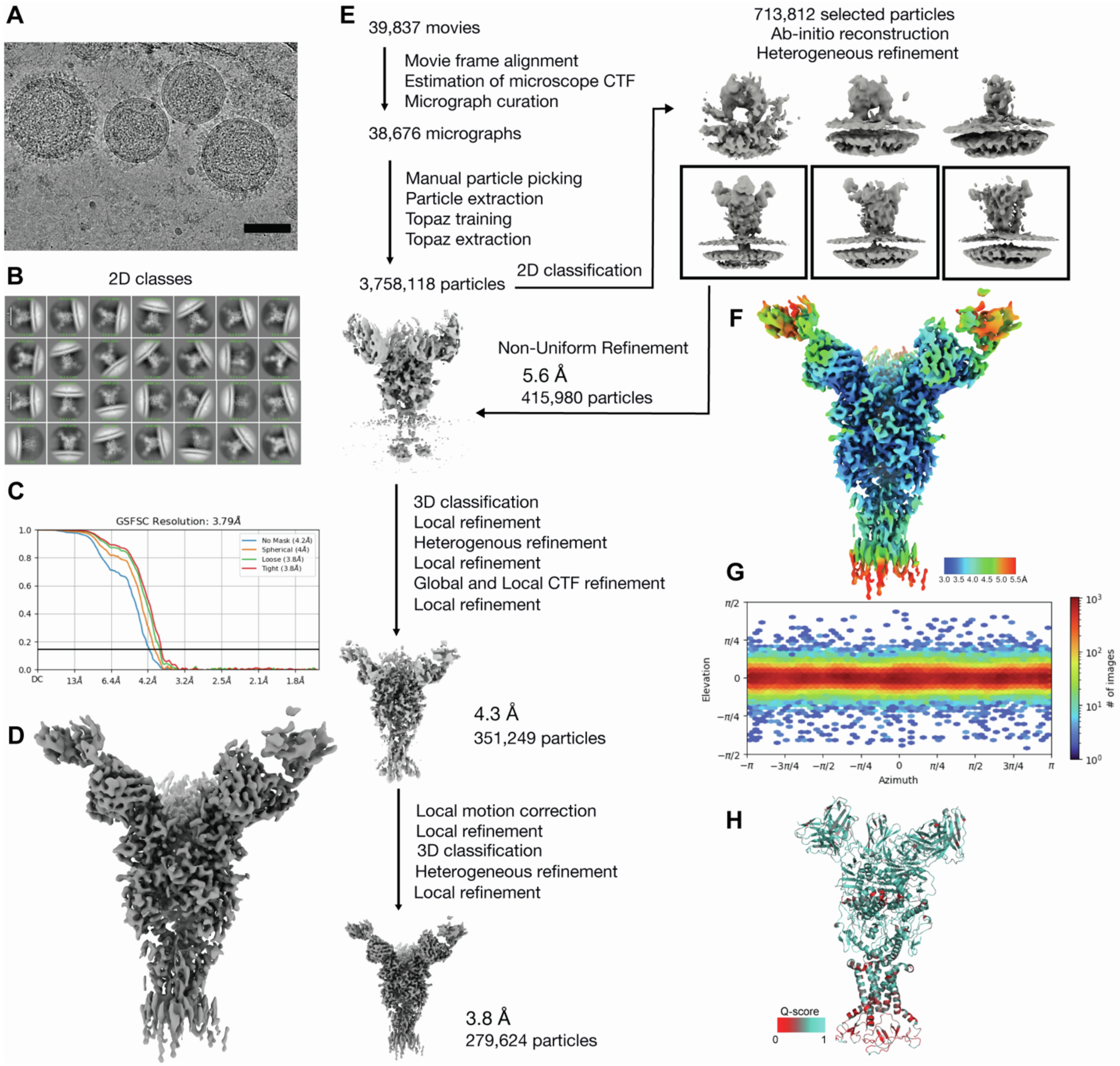

Figure S17. **cryo-EM data processing of the J199Fab-bound JUNV GPC-pseudotyped virus particles dataset, related to Figure 4**. (A) Representative cryo-electron micrograph of J199Fab-bound JUNV PV. Scale bar: 100 nm. (B) 2D classes of the J199Fab-bound JUNV GPC embedded in the viral membrane. (C) Gold-standard Fourier shell correlation curve with the 0.143 threshold indicated by a horizontal black line. (D) Sharpened final map at 3.8 Å. (E) cryo-EM data processing workflow. CTF = contrast transfer function. (F) Local resolution map for the JUNV GPC trimer. (G) The particle orientation density plot calculated using cryoSPARC is shown below the local resolution map. (H) J199Fab-bound JUNV GPC model colored by Q-score values, which indicate the correlation between the built atomic model and the corresponding cryo-EM map[3].

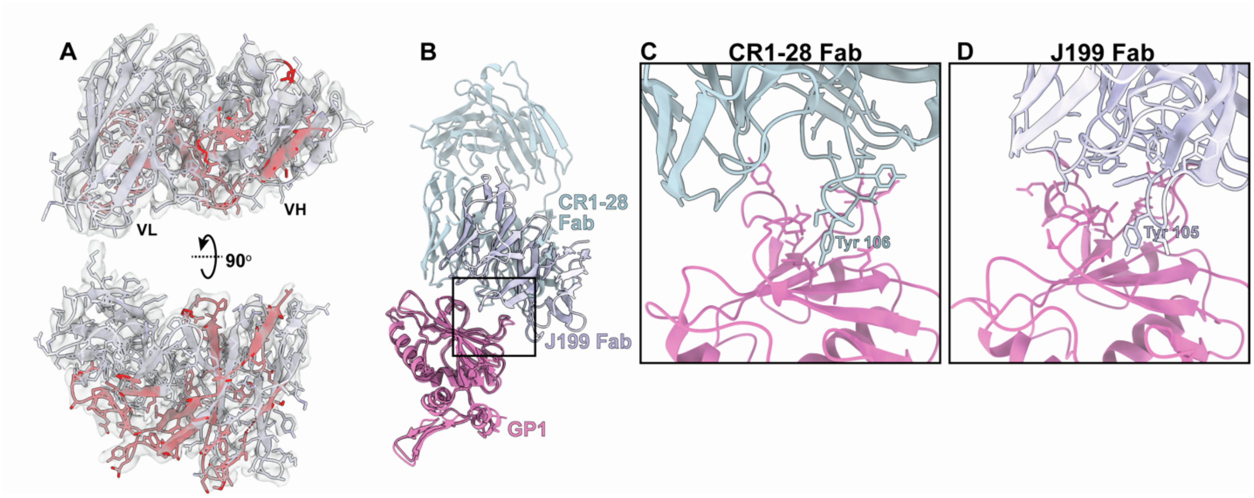

Figure S18. **Comparison of JUNV GP1-Fab interfaces, related to Figure 4**. (A) J199Fab model fit in cryo-EM density map. Segments not identified by bottom-up mass spectrometry are colored in red. (B) Overlay of GP1-CR1-28Fab and GP1-J199Fab. (C) View of GP1-CR1-28Fab interface. (D) View of GP1-J199Fab interface.

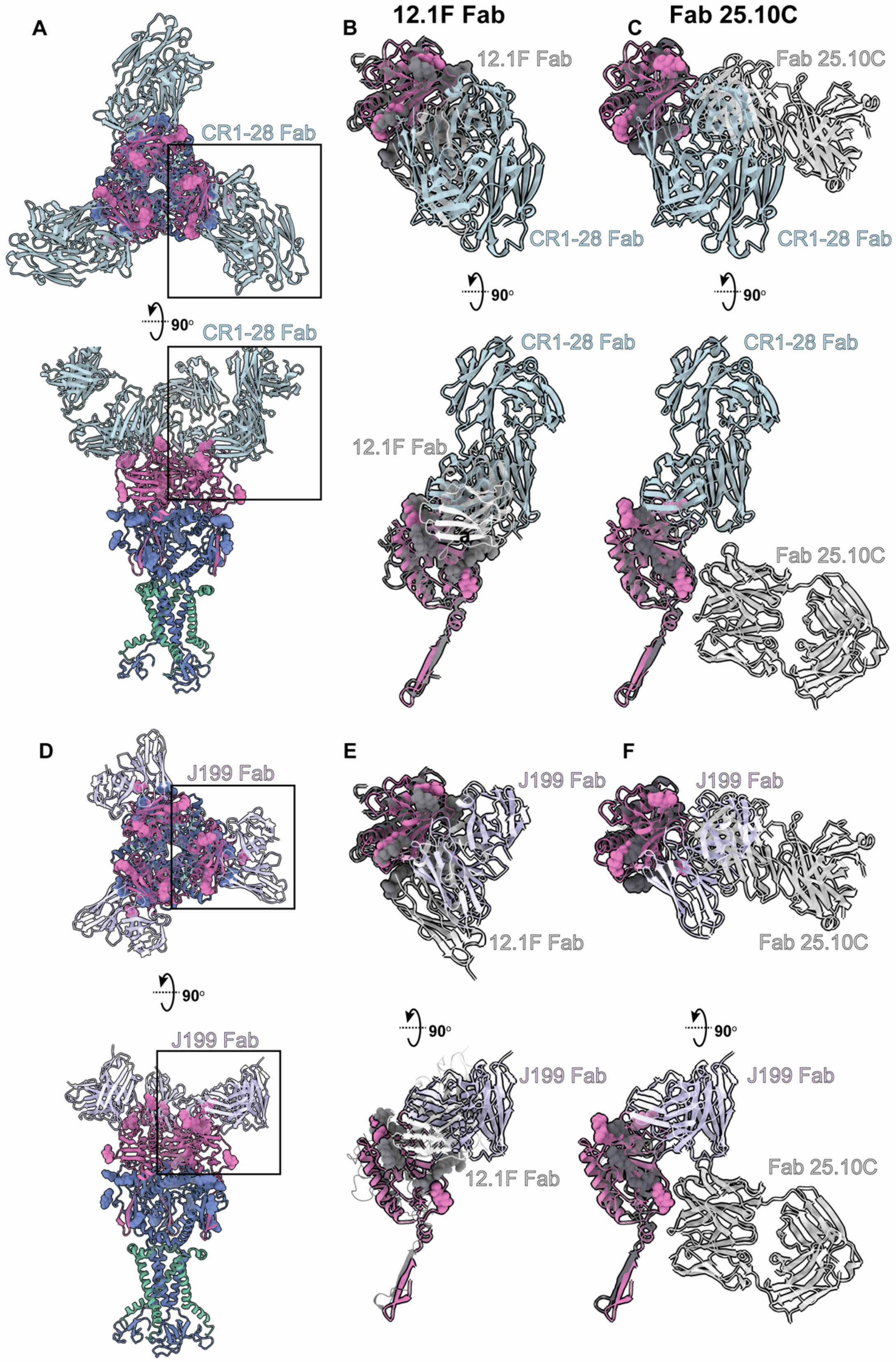

A
CR1-28 Fab
90°
CR1-28 Fab
B
12.1F Fab
12.1F Fab
CR1-28 Fab
90°
CR1-28 Fab
12.1F Fab
C
Fab 25.10C
Fab 25.10C
CR1-28 Fab
90°
CR1-28 Fab
Fab 25.10C
D
J199 Fab
90°
J199 Fab
E
J199 Fab
12.1F Fab
90°
J199 Fab
12.1F Fab
F
J199 Fab
Fab 25.10C
90°
J199 Fab
Fab 25.10C

Figure S19. **Comparison of JUNV and LASV GP1-Fab interfaces, related to Figure 4**. (A) Top and side views of the JUNV GPC bound to CR1-28Fab, glycans shown as space-fill models. (B) Overlay of JUNV GP1-CR1-28Fab and LASV GP1-12.1F Fab[4] (PDB: 8EJH). (C) Overlay of JUNV GP1-CR1-28Fab and LASV GP1-Fab 25.10C[5] (PDB: 7TYV). (D) Top and side views of the JUNV GPC bound to J199, glycans shown as space-fill models. (E) Overlay of JUNV GP1-J199Fab and LASV GP1-12.1F Fab (PDB: 8EJH). (F) Overlay of JUNV GP1-J199Fab and LASV GP1-Fab 25.10C (PDB: 7TYV).

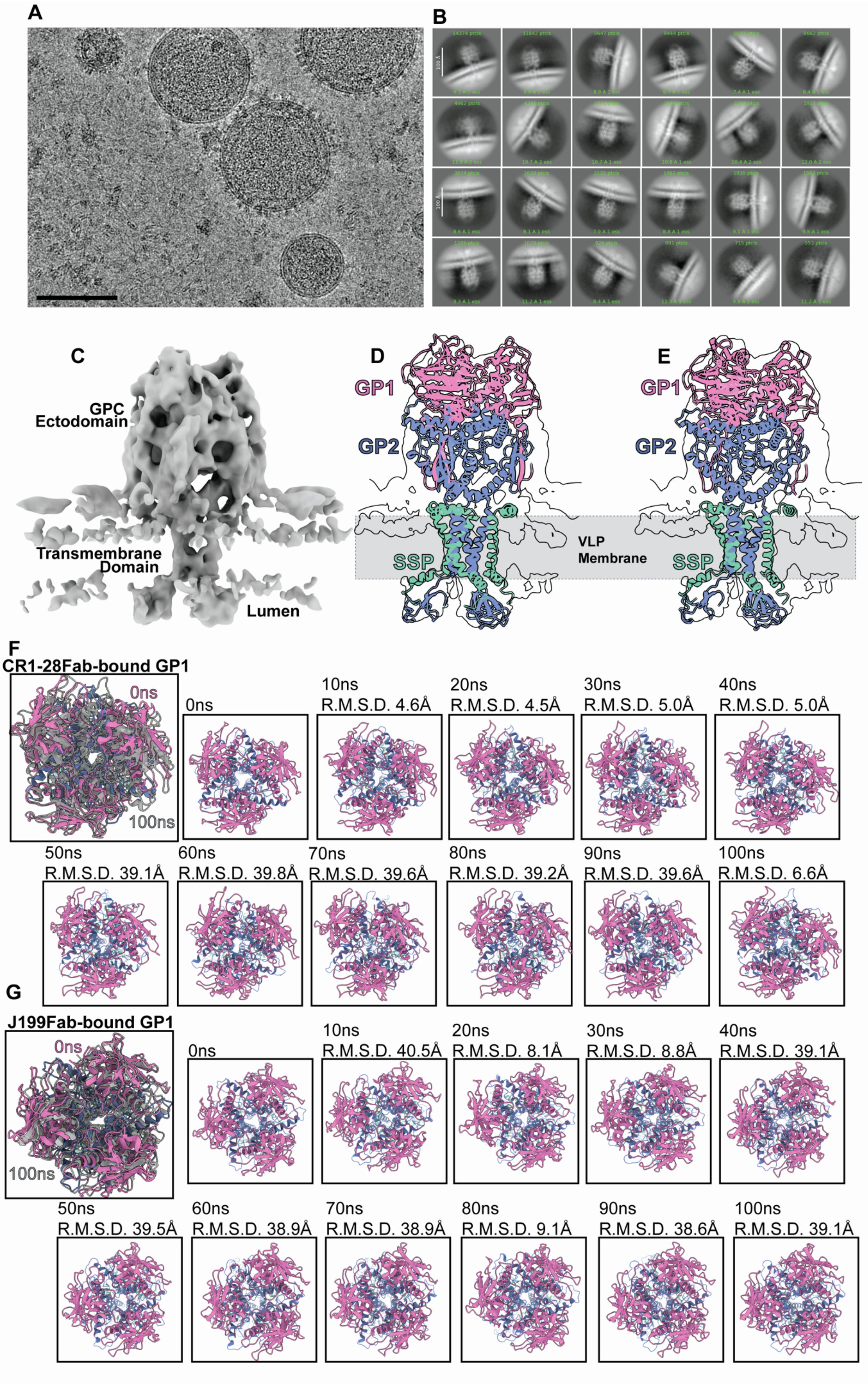
A
B
C
GPC
Ectodomain
Transmembrane
Domain
Lumen
D
GP1
GP2
SSP
VLP
Membrane
E
GP1
GP2
SSP
F
CR1-28Fab-bound GP1
0ns
100ns
0ns
10ns
R.M.S.D. 4.6Å
20ns
R.M.S.D. 4.5Å
30ns
R.M.S.D. 5.0Å
40ns
R.M.S.D. 5.0Å
50ns
R.M.S.D. 39.1Å
60ns
R.M.S.D. 39.8Å
70ns
R.M.S.D. 39.6Å
80ns
R.M.S.D. 39.2Å
90ns
R.M.S.D. 39.6Å
100ns
R.M.S.D. 6.6Å
G
J199Fab-bound GP1
0ns
100ns
0ns
10ns
R.M.S.D. 40.5Å
20ns
R.M.S.D. 8.1Å
30ns
R.M.S.D. 8.8Å
40ns
R.M.S.D. 39.1Å
50ns
R.M.S.D. 39.5Å
60ns
R.M.S.D. 38.9Å
70ns
R.M.S.D. 38.9Å
80ns
R.M.S.D. 9.1Å
90ns
R.M.S.D. 38.6Å
100ns
R.M.S.D. 39.1Å

Figure S20. **cryo-EM and computational analysis of the unbound JUNV GPC, related to Figures 1 and 4**. (A) Representative cryo-EM micrograph of unbound JUNV GPC PV; scale bar 100 nm. (B) 2D classes of unbound JUNV GPC embedded in PV membrane. (C) cryo-EM map of the JUNV GPC installed on a PV membrane at sigma level 0.45. (D) Model of JUNV GPC from CR1-28Fab-bound structure fit into cryo-EM density of unbound JUNV GPC. (E) Model of JUNV GPC from J199Fab-bound structure fit into cryo-EM density of unbound JUNV GPC. (F-G) 100ns molecular dynamics simulation of the JUNV GPC in its (F) CR1-28 bound state and (G) J199 bound state. Alignment was conducted for each GPC by one GP1 domain. R.M.S.D. values were computed for GP1 domains relative to the input state.

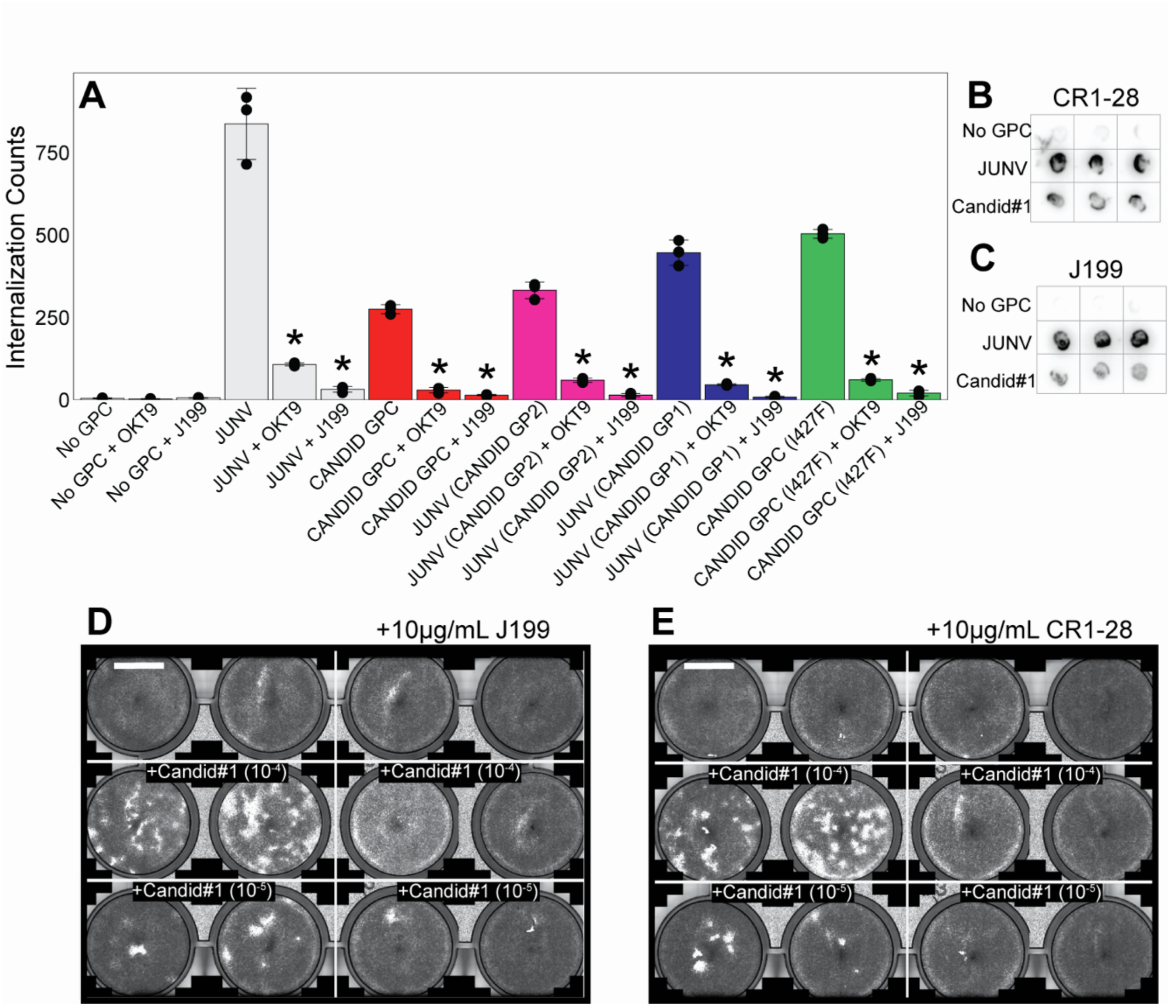

Figure S21. Assessing neutralization of JUNV and Candid#1 by CR1-28 and J199, related to Figure 4. (A) Quantification of GFP signal for mutant JUNV GPC PVs bearing the Candid#1 mutations indicated with 100 μg/mL OKT9 and 10 μg/mL J199. Data is represented as mean total GFP counts for each condition +/- standard deviation, n=3 technical replicates. *p-value <0.01 comparing OKT9 and J199 inhibited internalization of each pseudotype relative to uninhibited internalization determined by Welch's T-test. (B-C) Dot blots showing chemiluminescence signal for binding of primary antibody CR1-28 (A) or J199 (B) against PV media. (D-E) Titer assays depicting Candid#1 infection in the presence of 10 μg/mL J199 (D) or 10 μg/mL CR1-28 (E); scale bar, 10 mm.

Table S1. cryo-EM data collection, image analysis, modeling, refinement, and validation statistics for the CR1-28Fab-GPC structure, related to Figure 1.

**Dataset**	**JUNV GPC + CR1-28Fab on MLV membrane**
**Map refinement**	Focused on JUNV GPC + CR1-28Fab
**Data collection and processing**	
**Microscope**	Titan Krios
**Voltage (keV)**	300
**Detector**	K3
**Nominal magnification**	81,000
**Data Acquisition Software**	SerialEM
**Electron dose (e^{-}/Å^{2})**	40
**Pixel Size (Å)**	1.114
**Defocus range (μm)**	-1.00 to -2.50
**Number of movies (#)**	39,237
**Number of particles**	397,489
**Symmetry imposed**	C3
**Resolution (Å)**	3.8
**FSC threshold**	0.143
**Refinement**	
**Initial model used (PDB code)**	*7PUY, 5W1K*
**Non-hydrogen atoms**	21,744
**Protein residues**	2,739
**R.M.S. deviations**	
**Bond lengths (Å)**	0.003
**Bond angles (°)**	0.07
**Validation**	
**MolProbity score**	2.41
**Clashscore**	8.42
**Poor rotamers (%)**	3.28
**Ramachandran (%)**	
**Favored**	95.61
**Allowed**	4.39
**Disallowed**	0
**Fit to map (CC_{mask})**	0.68
**Accession codes**	
**EMDB (maps)**	EMD-48221
**PDB (model)**	9MEW

Table S2. cryo-EM data collection, image analysis, modeling, refinement, and validation statistics for the J199Fab-GPC structure, related to Figure 4.

**Dataset**	**JUNV GPC + J199Fab on MLV membrane**
**Map refinement**	Focused on JUNV GPC + J199Fab
**Data collection and processing**	
**Microscope**	Titan Krios
**Voltage (keV)**	300
**Detector**	K3
**Nominal magnification**	105,000
**Data Acquisition Software**	Leginon
**Electron dose (e^{-}/Å^{2})**	40
**Pixel Size (Å)**	0.826
**Defocus range (µm)**	-0.6 to -2.0
**Number of movies (#)**	39,837
**Number of particles**	279,624
**Symmetry imposed**	C3
**Resolution (Å)**	3.8
**FSC threshold**	0.143
**Refinement**	
**Initial model used (PDB code)**	***9MEW***
**Non-hydrogen atoms**	16,989
**Protein residues**	2,037
**R.M.S. deviations**	
**Bond lengths (Å)**	0.004
**Bond angles (°)**	1.09
**Validation**	
**MolProbity score**	2.58
**Clashscore**	12.22
**Poor rotamers (%)**	4.85
**Ramachandran (%)**	
**Favored**	92.64
**Allowed**	7.36
**Disallowed**	0
**Fit to map (CC_{mask})**	0.74
**Accession codes**	
**EMDB (maps)**	EMD-48781
**PDB (model)**	9N0D

Table S3. Experimental design for evaluation of OKT9 and huJ199 treatment in the hTfR1 mouse JUNV challenge model, related to Figure 4.

No./ Group	Group	Infected Y or N	Treatment	Dose & Route	Observations & Testing
10	1	Y	400 µg OKT9	0.1 mL IP administration at 1 h pre and 3 d post-infection	Observed for weight loss and mortality through day 29
9	2	Y	100 µg OKT9		
12	3	Y	PBS Placebo		
9	4	Y	200 µg huJ199		
3	5	N	Normal controls for weight change		

Table S4: Pairwise comparison p-values of OKT9 and huJ199 treatments against Placebo in the hTfR1 mouse JUNV challenge model, related to Figure 4.

**Treatment Group**	**Log-rank Test (Mantel-Cox) p-value**
Placebo	—
huJ199	0.0002
OKT9 High (400 µg)	0.0680
OKT9 Low (400 µg)	0.0624

Table S5. Plasmids used in this study, related to Key Resources Table.

Name	Description	Vector Backbone	Gene
MLV gag/pol	MLV gag/pol	CMV	MLV gag/pol
eGFP	GFP reporter	pQCXIX	eGFP
NLS-eGFP	GFP reporter with nuclear localization signal	pQCXIX	eGFP
JUNV GPC	Codon-optimized JUNV GPC sequence from Junin virus strain MC2 (D10072)	pcDNA 3.1+	JUNV GPC
MACV GPC	Codon optimized MACV GPC sequence from Machupo virus strain Carvallo (NC_005078)	pcDNA 3.1+	MACV GPC
TCRV GPC	TCRV GPC laboratory-adapted strain from S. Kunz, University of Lausanne, Switzerland, with a deletion spanning residues121–132 and substitutions at three residues (I134A, G418S and E458R) compared to TCRV (NC_004293) [6]	pcDNA 3.1+	TCRV GPC
TCRV*	Residues 86-241 of the laboratory-adapted TCRV GP1 cloned into MACV GPC backbone	pcDNA 3.1+	TCRV/MACV hybrid GPC
CR1-28 LC	CR1-28 antibody light chain	pVRC8400	CR1-28 Light-Chain
CR1-28 HC	CR1-28 antibody heavy chain	pVRC8400	CR1-28 Light-Chain
CR1-28 HC Fab-GFP	CR1-28 antibody heavy chain fab region with C-terminal Baojin GFP fusion and 7-residue his tag	pVRC8400	CR1-28 Heavy Chain Fab
CANDID GPC	JUNV virus strain Candid #1 segment S, complete sequence (FJ969442.1)	pcDNA 3.1+	CANDID GPC

Table S6. GPC sequence variants reported in this study, related to Key Resources Table.

Name:	Reference vector:	Mutation(s):	Mutation Region(s):	Functional Impact:
CANDID GPC	JUNV GPC	K109Q, E121Q, Y157H, T168A, I184V, E186G, S206P, D209N, F427I, T446S	GP1, GP2	Moderate reduction of signal
JUNV (CANDID GP1)	JUNV GPC	K109Q, E121Q, Y157H, T168A, I184V, E186G, S206P, D209N,	GP1	Moderate reduction of signal
JUNV (CANDID GP2)	JUNV GPC	F427I, T446S	GP2	Moderate reduction of signal
CANDID GPC I427F	CANDID GPC	I427F	Transmembrane of GP2	Moderate reduction of signal
JUNV (TCRV GP1)	JUNV GPC	TCRV GP1	GP1	Not TfR1-dependent signal
TCRV (JUNV GP1)	TCRV GPC	JUNV GP1	GP1	Not detectable
F433I	JUNV GPC	F433I	Transmembrane of GP2	Moderate reduction of signal
F433A	JUNV GPC	F433A	Transmembrane of GP2	Moderate reduction of signal
Q16A	JUNV GPC	Q16A	SSP	Function retained
E17K_K417E	JUNV GPC	E17K_K417E	SSP, GP2	Significant reduction of signal
F14Y	JUNV GPC	F14Y	SSP	Function retained
F14W	JUNV GPC	F14W	SSP	Moderate reduction of signal
K33A	JUNV GPC	K33A	Transmembrane of SSP	Function depleted
K33F	JUNV GPC	K33F	Transmembrane of SSP	Function depleted

Table S7: Amino acid sequences of plasmids used in this study, related to Key Resources Table.

Plasmid	Sequence
MLV gagpol	MGQTVTTPLSLTLGHWKDVERIAHNQSVDVKKRRWVTFCSAEWPTFNVGWPRDGTFN<br>RDLITQVKIKVFSPGPHGHPDQVPYIVTWEALAFDPPPWVKPFVHPKPPPPLPPSAPSL<br>PLEPPRSTPPRSSLYPALTPSLGAKPKPQVLSDSGGPLIDLLTEDPPPYRDPRPPPSDRD<br>GNGGEATPAGEAPDPSPMASRLRGRREPPVADSTTSQAFPLRAGGNGQLQYWPFSSS<br>DLYNWKNNNPSFSEDPGKLTALIESVLITHQPTWDDCQQLLGTLLTGEEKQRVLLEARK<br>AVRGDDGRPTQLPNEVDAAFPLERPDWDYTTQAGRNHLVHYRQLLLAGLQNAGRSPT<br>NLAKVKGITQGPNESPSAFLERLKEAYRRYTPYDPEDPGQETNVSMSFIWQSAPDIGRK<br>LERLEDLKNKTLGDLVREAEKIFNKRETPEEREERIRRETEEKEERRRTEDEQKEKERD<br>RRRHREMSKLLATVVSGQKQDRQGGERRRSQLDRDQCAYCKEKGHWAKDCPKKPRG<br>PRGPRPQTSLLTLDDXGGQGQEPPPEPRITLKVGGQPVTFLVDTGAQHSVLTQNPGPL<br>SDKSAWVQGATGGKRYRWTTDRKVHLATGKVTHSFLHVPDCPYPLLGRDLLTKLKAQI<br>HFEGSGAQVMGPMGQPLQVLTLNIEDEYRLHETSKEPDVSLGSTWLSDFPQAWAETG<br>GMGLAVRQAPLIIPLKATSTPVSIKQYPMSQEARLGIKPHIQRLLDQGILVPCQSPWNTPL<br>LPVKKPGTNDYRPVQDLREVNKRVEDIHPTVPNPYNLLSGLPPSHQWYTVLDLKDAFF<br>CLRLHPTSQPLFAFEWRDPEMGISGQLTWTRLPQGFKNSPTLFDEALHRDLADFRIQHP<br>DLILLQYVDDLLLAATSELDCQQGTRALLQTLGNLGYRASAKKAQICQKQVKYLGYLLKE<br>GQRWLTEARKETVMGQPTPKTPRQLREFLGTAGFCRLWIPGFAEMAAPLYPLTKTGTLF<br>NWGPDQQKAYQEIKQALLTAPALGLPDLTKPFELFVDEKQGYAKGVLTQKLGPWRRPVA<br>YLSKKLDPVAAGWPPCLRMVAAIAVLTKDAGKLTMGQPLVILAPHAVEALVKQPPDRWLS<br>NARMTHYQALLLDTDRVQFGPVVALNPATLLPLPEEGLQHNCLDILAEAHGTRPDLTDQ<br>PLPDADHTWYTDGSSLLQEGQRKAGAAVTTETEVIWAKALPAGTSAQRAELIALTQALK<br>MAEGKKLNVYTDSRYAFATAHIHGEIYRRRGLLTSEGKEIKNKDEILALLKALFLPKRLSIIH<br>CPGHQKGHSAEARGNRMADQAARKAAITETPDTSTLLIENSSPYTSEHFHYTVTDIKDL<br>TKLGAIYDKTKKYWVYQGKPVMPDQFTFELLDFLHQLTHLSFSKMKALLERSHSPYYML<br>NRDRTLKNITETCKACAQVNASKSAVKQGTRVRGHRPGTHWEIDFTEIKPGLYGYKYLL<br>VFIDTFSGWIEAFPTKKETAKVVTKKLLEEIFPRFGMPQVLGTDNGPAFVSKVSQTVADL<br>LGIDWKLHCAYRPQSSGQVERMNRTIKETLTKLTLATGSRDWVLLLPLALYRARNTPGP<br>HGLTPYEILYGAPPPLVNFPDPDMTRVTNSPSLQAHLQALYLVQHEVWRPLAAAYQEQL<br>DRPVVPHPYRVGDTVWVRRHQTKNLEPRWKGPYTVLLTTPTALKVDGIAAWIHAAHVK<br>AADPGGGPSSRLTWRVQRSQNPLKIRLTREAP
eGFP	SKGEELFTGVVPILVELDGDVNGHKFSVSGEGEGDATYGKLTLKFICTTGKLPVPWPTLV<br>TTLTYGVQCFSRYPDHMKQHDFFKSAMPEGYVQERTIFFKDDGNYKTRAEVKFEGDTL<br>VNRIELKGIDFKEDGNILGHKLEYNYNSHNVYIMADKQKNGIKVNFKIRHNIEDGSVQLAD<br>HYQQNTPIGDGPVLLPDNHYLSTQSALSKDPNEKRDHMVLLEFVTAAGITLGMDELYK
NLS-GFP	MAPKKKRKVMVSKGEELFTGVVPILVELDGDVNGHKFSVSGEGEGDATYGKLTLKFICT<br>TGKLPVPWPTLVTTLTYGVQCFSRYPDHMKQHDFFKSAMPEGYVQERTIFFKDDGNYK<br>TRAEVKFEGDTLVNRIELKGIDFKEDGNILGHKLEYNYNSHNVYIMADKQKNGIKVNFKIR<br>HNIEDGSVQLADHYQQNTPIGDGPVLLPDNHYLSTQSALSKDPNEKRDHMVLLEFVTAA<br>GITLGMDELYK
JUNV GPC	MGQFISFMQEIPTFLQEALNIALVAVSLIAIIKGVVNLYKSGLFQFFVFLALAGRSCTEEAFK<br>IGLHTEFQTVSFSMVGLFSNNPHDLPLLCTLNKSHLYIKGGNASFKISFDDIAVLLPEYDVI<br>IQHPADMSWCSKSDDQIWLSQWFMNAVGHDWYLDPPFLCRNRTKTEGFIFQVNTSKT<br>GINENYAKKFKTGMHHLYREYPDSCLDGKLCLMKAQPTSWPLQCPLDHVNTLHFLTRG<br>KNIQLPRRSLKAFFSWSLTDSSGKDTPGGYCLEEWMLVAAKMKCFGNTAVAKCNLNHD<br>SEFCDMLRLFDYNKNAIKTLNDETKKQVNLMGQTINALISDNLLMKNKIRELMSVPYCNY<br>TKFWYVNHTLSGQHSLPRCWLIKNNSYLNISDFRNDWILESDFLISEMLSKEYSDRQGK

	TPLTLVDICFWSTVFFTASLFLHLVGIPTHRHIRGEACPLPHRLNSLGGCRCGKYPNLKKP TVWRRGH
MACV GPC	MGQLISFFQEIPVFLQEALNIALVAVSLIAVIKGIINLYKSGLFQFIFFLLLAGRSCSDGTFKI GLHTEFQSVTLTMQRLLANHSNELPSLCMLNNSFYYMRGGVNTFLIRVSDISVLMKEYD VSIYEPEDLGNCLNKSDSSWAIHWFSNALGHDWLMDPPMLCRNKTKKEGSNIQFNISK ADDARVYGKKIRNGMRHLFRGFHDPCEEGKVCYLTINQCGDPSSFDYCGVNHLSKCQF DHVNTLHFLVRSKTHLNFERSLKAFFSWSLTDSSGKDMPGGYCLEEWMLIAAKMKCFG NTAVAKCNQNHDSEFCDMLRLFDYNKNAIKTLNDESKKEINLLSQTVNALISDNLLMKNKI KELMSIPYCNYTKFWYVNHTLTGQHTLPRCWLIRNGSYLNTSEFRNDWILESDHLISEM LSKEYAERQGKTPITLVDICFWSTIFFTASLFLHLVGIPTHRHLKGEACPLPHKLDSFGGC RCGKYPRLKKPTIWHKRH
TCRV GPC	MGQFISFMQEIPIFLQEALNIALVAVSLICIVKGLVNLYRCGLFQLMVFLVLAGRSCSEETF KIGMHTKFQEVSLSLSALLTNQSHELPMLCLANKTHLYLKSGRSSFKINIDSVTVLTRSAD VFVHSPKLGSCFESDEEWVVAWWIEAIGHRWDQDPGLLCRNKTKTEGKLIQINISRADG NVHYGWRLKNGLDHIYRGREEPCFEGEQCLIKIQPEDWPTDCKADHTNTFRFLSRSQK SIAVGRTLKAFFSWSLTDPLGNEAPGGYCLEKWMLVASELKCFGNTAIAKCNQNHDSEF CDMLRLFDYNKNAIKTLNEETKTRVNVLSHTINALISDNLLMKNKIRELMSVPYCNYTRF WYVNHTLSGQHSLPRCWMIRNNSYLNSSEFRNEWILESDFLISEMLSKEYSERQGRTPI TLVDICFWSTVFFTSTLFLHLIGFPTHRHIRGEGCPLPHRLNSMGGCRCGKYLPLKKPTI WHRRH
TCRV*	MGQFISFMQEIPIFLQEALNIALVAVSLICIVKGLVNLYRCGLFQLMVFLVLAGRSCSEETF KIGMHTKFQEVSLSLSALLTNQSHELPMLCLANKTHLYLKSGRSSFKINIDSVTVLTRSAD VFVHSPKLGSCFESDEEWVVAWWIEAIGHRWDQDPGLLCRNKTKTEGKLIQINISRADG NVHYGWRLKNGLDHIYRGREEPCFEGEQCLIKIQPEDWPTDCKADHTNTFRFLSRSQK SIAVGRTLKAFFSWSLTDPLGNEAPGGYCLEKWMLVASELKCFGNTAIAKCNQNHDSEF CDMLRLFDYNKNAIKTLNEETKTRVNVLSHTINALISDNLLMKNKIRELMSVPYCNYTRF WYVNHTLSGQHSLPRCWMIRNNSYLNSSEFRNEWILESDFLISEMLSKEYSERQGRTPI TLVDICFWSTVFFTSTLFLHLIGFPTHRHIRGEGCPLPHRLNSMGGCRCGKYLPLKKPTI WHRRH
CR1-28 LC	MDAMKRGLCCVLLLCGAVFVSPSASDIQMTQSPSTLSASVGDRVTITCRASQSIDNWLA WYQQKPGKAPKLLIYTASRLESGVPSRFSGSGSGTEFTLTISSLQPDDFATYYCQHRTF GQGTKVEIKRTVAAPSVFIFPPSDEQLKSGTASVVCLLNNFYPREAKVQWKVDNALQSG NSQESVTEQDSKDSTYSLSSTLTLSKADYEKHKVYACEVTHQGLSSPVTKSFNRGEC
CR1-28 HC	MDAMKRGLCCVLLLCGAVFVSPSASQVQLVESGGGVVQPGRSLRLSCAASGFTFSSS AMHWVRQAPGKGLEWVAVIWSDGSNENYADSVKGRFTISRDNSKNTLYLQMSSLRAE DTAVYYCATDKTYVSGYTSTWYYFNYWGQGTLVTVSGASTKGPSVFPLAPSSKSTSGG TAALGCLVKDYFPEPVTVSWNSGALTSGVHTFPAVLQSSGLYSLSSVVTVPSSSLGTQT YICNVNHKPSNTKVDKRVEPKSCDRTHTCPPCPAPELLGGPSVFLFPPKPKDTLMISRT PEVTCVVVDVSHEDPEVKFNWYVDGVEVHNAKTKPREEQYNSTYRVVSVLTVLHQDW LNGKEYKCKVSNKALPAPIEKTISKAKGQPREPQVYTLPPSREEMTKNQVSLTCLVKGF YPSDIAVEWESNGQPENNYKTTPPVLDSDGSFFLYSKLTVDKSRWQQGNVFSCSVMHE ALHNHYTQKSLSLSPGK
CR1-28 HC Fab(-GFP)	MDAMKRGLCCVLLLCGAVFVSPSASQVQLVESGGGVVQPGRSLRLSCAASGFTFSSS AMHWVRQAPGKGLEWVAVIWSDGSNENYADSVKGRFTISRDNSKNTLYLQMSSLRAE DTAVYYCATDKTYVSGYTSTWYYFNYWGQGTLVTVSGASTKGPSVFPLAPSSKSTSGG TAALGCLVKDYFPEPVTVSWNSGALTSGVHTFPAVLQSSGLYSLSSVVTVPSSSLGTQT YICNVNHKPSNTKVDKRVEPKSCGGGGSGGGGSGGGGSGGGGSMVSKGEEENMAS TPFKFQLKGTINGKSFTVEGEGEGNSHEGSHKGKYVCTSGKLPMSWAALGTTFGYGM KYYTKYPSGLKNWFREVMPGGFTYDRHIQYKGDGSIHAKHQHFMKNGTYHNIVEFTGQ DFKENSPVLTGDMNVSLPNEVPQIPRDDGVECPVTLLYPLLSDKSKYVEAHQYTICKPL HNQPAPDVPYHWIRKQYTQSKDDAERDHICQSETLEAHLKGMDELYKGSGGSGHHH HHHHH

CANDID GPC	MGQFISFMQEIPTFLQEALNIALVAVSLIAIIKGVVNLYKSGLFQFFVFLALAGRSCTEEAFK<br>IGLHTEFQTVSFSMVGLFSNNPHDLPLLCTLNKSHLYIKGGNASFQISFDDIAVLLPQYDV<br>IIQHPADMSWCSKSDDQIWLSQWFMNAVGHDWHLDPPFLCRNRAKTEGFIFQVNTSKT<br>GVNGNYAKKFKTGMHHLYREYPDPCLNGKLCLMKAQPTSWPLQCPLDHVNTLHFLTR<br>GKNIQLPRRSLKAFFSWSLTDSSGKDTPGGYCLEEWMLVAAKMKCFGNTAVAKCNLNH<br>DSEFCDMLRLFDYNKNAIKTLNDETKKQVNLMGQTINALISDNLLMKNKIRELMSVPYCN<br>YTKFWYVNHTLSGQHSLPRCWLIKNNSYLNISDFRNDWILESDFLISEMLSKEYSDRQG<br>KTPLTLVDICIWSTVFFTASLFLHLVGIPSHRHIRGEACPLPHRLNSLGGCRCGKYPNLKK<br>PTVWRRGH

References

1. Katoh, K., and Standley, D.M. (2013). MAFFT Multiple Sequence Alignment Software Version 7: Improvements in Performance and Usability. Mol Biol Evol *30*, 772–780. https://doi.org/10.1093/molbev/mst010.

2. Waterhouse, A.M., Procter, J.B., Martin, D.M.A., Clamp, M., and Barton, G.J. (2009). Jalview Version 2—a multiple sequence alignment editor and analysis workbench. Bioinformatics *25*, 1189–1191. https://doi.org/10.1093/bioinformatics/btp033.

3. Pintilie, G., Zhang, K., Su, Z., Li, S., Schmid, M.F., and Chiu, W. (2020). Measurement of Atom Resolvability in CryoEM Maps with Q-scores. Nat Methods *17*, 328–334. https://doi.org/10.1038/s41592-020-0731-1.

4. Perrett, H.R., Brouwer, P.J.M., Hurtado, J., Newby, M.L., Liu, L., Müller-Kräuter, H., Müller Aguirre, S., Burger, J.A., Bouhuijs, J.H., Gibson, G., et al. (2023). Structural conservation of Lassa virus glycoproteins and recognition by neutralizing antibodies. Cell Reports *42*, 112524. https://doi.org/10.1016/j.celrep.2023.112524.

5. Enriquez, A.S., Buck, T.K., Li, H., Norris, M.J., Moon-Walker, A., Zandonatti, M.A., Harkins, S.S., Robinson, J.E., Branco, L.M., Garry, R.F., et al. (2022). Delineating the mechanism of anti-Lassa virus GPC-A neutralizing antibodies. Cell Reports *39*, 110841. https://doi.org/10.1016/j.celrep.2022.110841.

6. Abraham, J., Corbett, K.D., Farzan, M., Choe, H., and Harrison, S.C. (2010). Structural basis for receptor recognition by New World hemorrhagic fever arenaviruses. Nature Structural & Molecular Biology *17*, 438. https://doi.org/10.1038/nsmb.1772.
